# Altered inflammatory mucosal signatures within their spatial and cellular context during active ileal Crohn’s disease

**DOI:** 10.1172/jci.insight.171783

**Published:** 2025-03-10

**Authors:** Vasantha L. Kolachala, Sushma Chowdary Maddipatla, Shanta Murthy, Yeonjoo Hwang, Anne F. Dodd, Garima Sharma, Sachith Munasinghe, Ranjit Singh Pelia, Suresh Venkateswaran, Murugadas Anbazhagan, Tarun Koti, Navdeep Jhita, Gaurav N. Joshi, Chrissy A. Lopez, Duke Geem, Hong Yin, David J. Cutler, Peng Qiu, Jason D. Matthews, Subra Kugathasan

**Affiliations:** 1 Division of Pediatric Gastroenterology, Department of Pediatrics & Pediatric Research Institute, Emory University School of Medicine & Children’s Healthcare of Atlanta;; 2 Department of Pathology, Children’s Healthcare of Atlanta;; 3 Department of Human Genetics; and; 4 Department of Biomedical Engineering, Georgia Institute of Technology and Emory University, Atlanta, Georgia, USA.

**Keywords:** Gastroenterology, Inflammation, Antigen, Cell migration/adhesion, Inflammatory bowel disease

## Abstract

Crohn’s disease (CD) involves a complex intestinal microenvironment driven by chronic inflammation. While single-cell RNA sequencing has provided valuable insights into this biology, the spatial context is lost during single-cell preparation of mucosal biopsies. To deepen our understanding of the distinct inflammatory signatures of CD and overcome the limitations of single-cell RNA sequencing, we combined spatial transcriptomics of frozen CD surgical tissue sections with single-cell transcriptomics of ileal CD mucosa. Coexpressed genes and cell-cell communication from single-cell analyses and factorized genes from spatial transcriptomics revealed overlapping pathways affected in inflamed CD, like antigen presentation, phagosome activity, cell adhesion, and extracellular matrix. Within the pathways, early epithelial cells showed evidence of significant changes in gene expression and subtype composition, while spatial mapping revealed the location of the events, particularly antigen presentation from epithelial cells in the base of the crypt. Furthermore, we identified early epithelial cells as a potential mediator of the MHC class II pathway during inflammation, which we validated by spatial transcriptomics cell subtype deconvolution. Therefore, the inflammation from CD appears to change the types of interactions detectable between epithelial cells with immune and mesenchymal cells, likely promoting the conditions for more macrophage infiltration into these inflammatory microdomains.

## Abstract 2

We integrated single-cell and spatial transcriptomics to identify cellular networks and coordinated molecules distinguishing active Crohn’s disease and assessed their spatial distribution on diseased tissues.

## Introduction

There is an unmet need to better define the molecular and cellular landscape of Crohn’s disease (CD), an inflammatory disease of the gastrointestinal tract that currently affects nearly 3 million individuals in North America (1–4). The heterogeneous nature of CD is related to the intricate and complex microenvironments of the intestine, where the immune and stromal compartments are separated from luminal contents and microbiome by an epithelial barrier. Factors including genetics, diet, smoking, and environmental conditions appear to contribute to the epithelium’s inability to maintain this barrier in CD, promoting a microenvironment of chronic inflammation with ongoing mucosal injury (5). Cellular signaling via receptor-ligand (RL) interactions between different cell types in the mucosa has been well established, with anti-TNF therapy being the most successful treatment to date that targets these processes. However, anti-TNF response remains transient in many patients with CD, with most patients eventually developing antibody resistance. Consequently, there is a need to better understand the mechanisms underlying persistent inflammation in CD.

Substantial gains in characterizing the cellular basis of inflammatory bowel disease (IBD) have been achieved using single-cell technologies (6–10). Despite this, the spatial context of cells in the mucosa is lost during single-cell preparation, making it difficult to localize the activity and interactions of cells. Thus, a more accurate view of the architectural, cellular, and molecular changes taking place in the inflamed ileal mucosa of CD can be made using spatial transcriptomics (ST) on resected surgical tissues in conjunction with single-cell RNA sequencing.

Here, we combine single-cell RNA-sequencing and ST approaches to enhance our view of cellular changes taking place in the ileal mucosa during active CD. Using cell-cell communication (11), coexpressed gene modules (12), and spatial factors (13, 14), we substantiated pathways represented during active CD and mapped their activity onto ileal resected tissues. Through our integration of single-cell and ST approaches, we uncover cell type activity and gene expression patterns varying between inflamed and noninflamed CD.

## Results

### Single-cell profiling of ileal mucosal cells derived from patients with Crohn’s.

**Figure 1:**
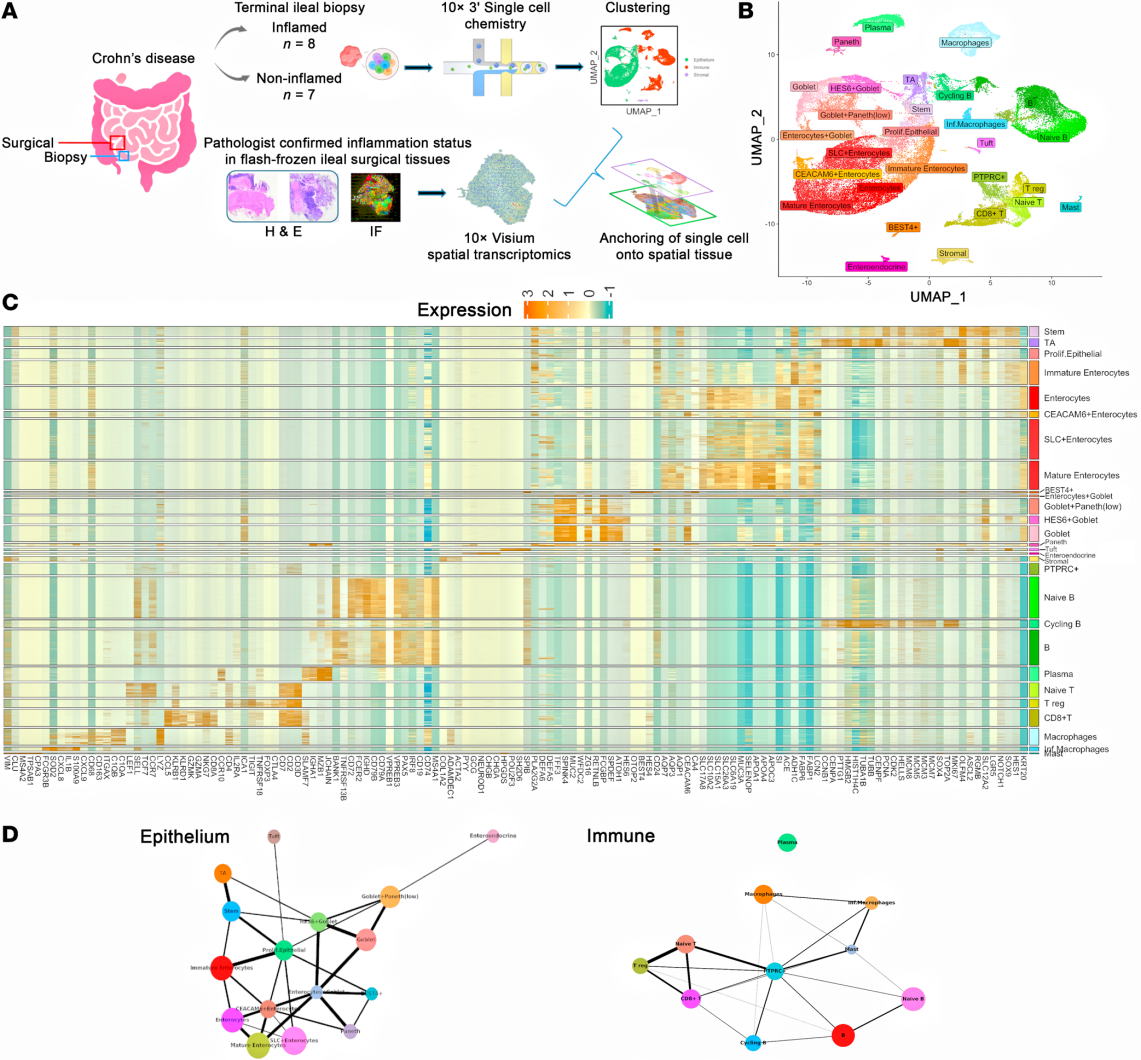
Experimental setup and single-cell transcriptomic analysis. (**A**) Schematic of ileal biopsy collection and single-cell and spatial transcriptomic analysis. (**B**) Uniform manifold approximation and projection (UMAP) of integrated and annotated single-cell transcriptomic data (*n* = 15), with 28 annotated cell types. (**C**) Heatmap showing the expression of cell type–specific marker genes. (**D**) Inferred trajectory of epithelial and immune differentiation.

To gain insight into the inflammation-associated cellular dynamics during CD, we performed single-cell RNA sequencing on ileal biopsies obtained from patients with CD, with *n* = 8 inflamed CD and *n* = 7 noninflamed CD, at the time of colonoscopy (Figure 1A and Table 1). After single-cell data preprocessing and quality control (Supplemental Figure 1, A–D; supplemental material available online with this article; https://doi.org/10.1172/jci.insight.171783DS1), the obtained 52,396 cells were clustered into 3 intestinal compartments — epithelial, immune, and stromal — that were annotated by transcriptional signature differences. Fine clustering and manual annotation based on canonical marker genes’ expression further identified 28 cell types (Supplemental Table 1). Of these cell types, 16 were epithelial, 11 were immune, and 1 was a stromal subpopulation (Figure 1, B and C). Next, we delineated relationships between cell types using trajectory analysis with partition-based graph abstraction (PAGA). This approach supported our manual annotations by quantifying the connection strengths between annotated cell types within epithelial and immune subpopulations (Figure 1D).

### Epithelial and B cell subtypes exhibit proportional and transcriptional changes associated with ileal mucosal inflammation during CD.

**Figure 2:**
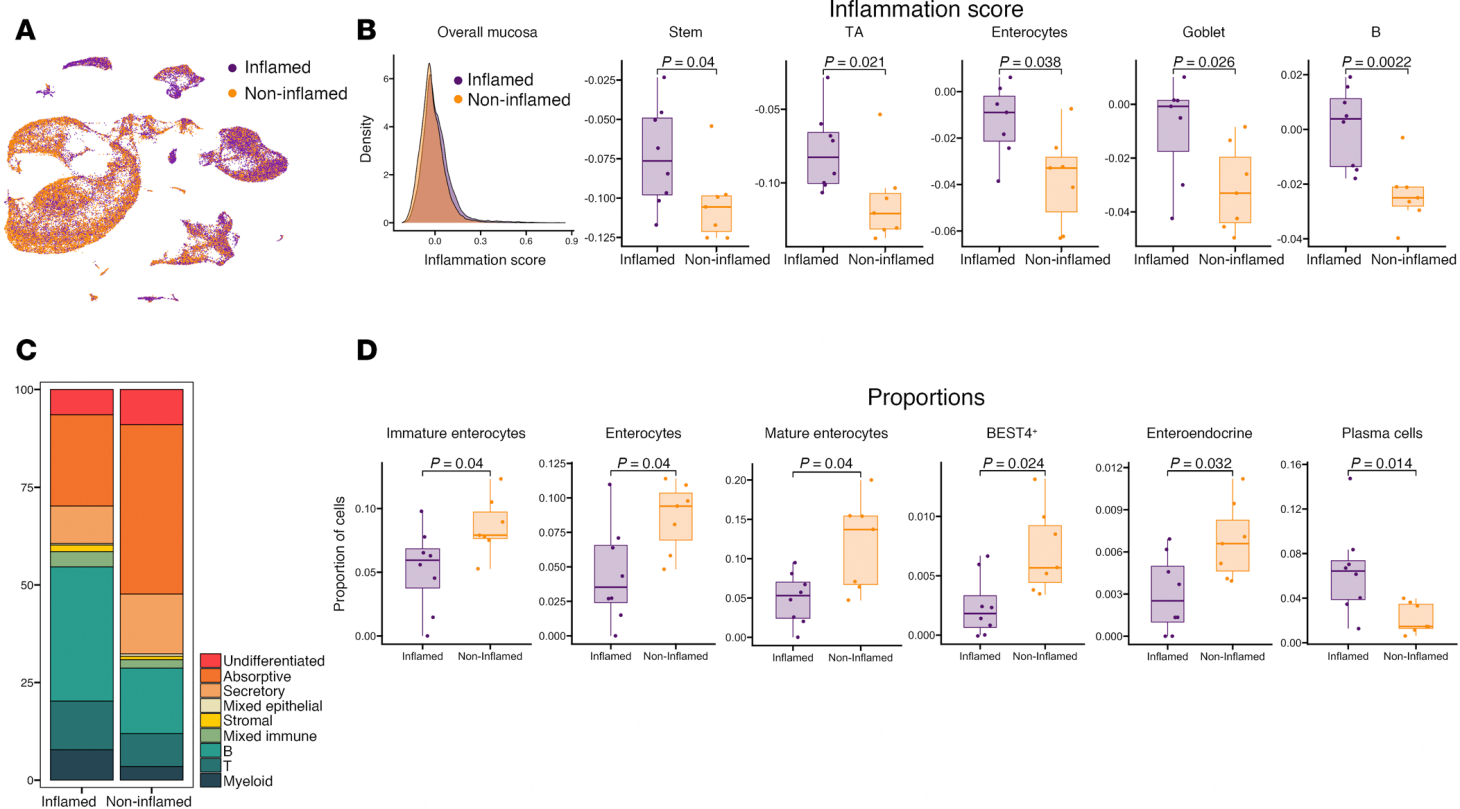
Cellular and transcriptional changes associated with mucosal inflammation in CD. (**A**) UMAP overlay colored by inflammation status. (**B**) Inflammation score density for inflamed and noninflamed separately in overall mucosa (leftmost plot) and box plots of selected cell types showing significant difference in inflammation score between the 2 groups (Wilcoxon’s rank sum test, *P* value < 0.05). (**C**) Bar plot showing cell lineage proportions between inflamed and noninflamed groups in overall mucosa. (**D**) Box plots of the selected cell types with significance (Student’s *t* test, *P* value < 0.05).Box plots show the interquartile range, median (line), and minimum and maximum (whiskers).

Using a list of published inflammation-associated genes, we evaluated inflammation scores across cells in inflamed and noninflamed CD (Figure 2A and Supplemental Figure 1E). We did not find global differences in this score between inflamed and noninflamed CD mucosa (Figure 2B). However, we detected a significant increase in inflammatory scores in the stem cells, transit-amplifying cells, goblet cells, and enterocytes in the epithelium, along with B cells in the immune compartment in inflamed CD. When examining cellular proportions between the noninflamed and inflamed mucosa, we observed a marked decrease in absorptive epithelial lineage and a substantial increase in immune populations (Figure 2C). Specifically, the relative proportions of absorptive epithelial cells such as immature enterocytes and enterocytes, mature enterocytes, BEST4^+^ cells, and enteroendocrine cells were significantly reduced, while the abundance of plasma cells, a B cell subtype, increased during inflammation (Figure 2D).

### Antigen presentation and cell adhesion molecules are promoted in early epithelial cells and lymphocyte subtypes during mucosal inflammation.

**Figure 3:**
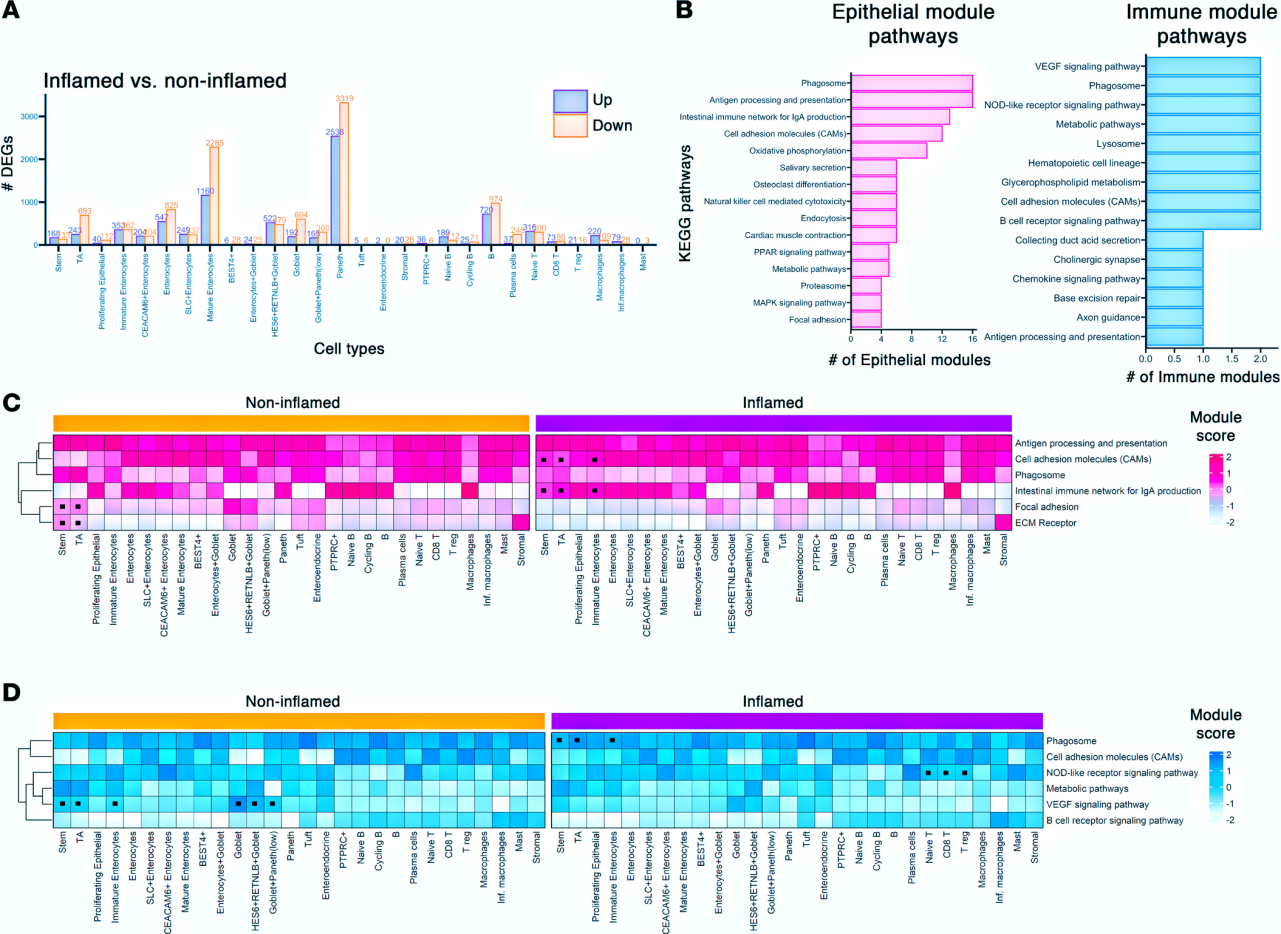
Antigen presentation and cell adhesion molecules are promoted during mucosal inflammation. (**A**) Bar plot of the number of significantly (adj. *P* value < 0.05) differentially expressed genes (DEGs) in inflamed (purple) versus noninflamed (orange) cells per cell type using the MAST algorithm. (**B**) Bar plots of the frequency of Kyoto Encyclopedia of Genes and Genomes (KEGG) pathways significantly (*P* value < 0.05) enriched in epithelial (pink) and immune (blue) cell type modules of the DEGs. (**C**) Heatmaps of key epithelial module scores across the cell types in inflamed and noninflamed groups. Dots represent enrichment. (**D**) Heatmaps of key immune module scores across the cell types in inflamed and noninflamed groups. Dots represent enrichment.

To further understand gene expression patterns during active inflammation in CD, we used gene modules to investigate coexpression of (DEGs) within each cell subtype. We first identified 11,480 unique significant DEGs within the cell types from the inflamed ileal mucosa, with Paneth cells and mature enterocytes exhibiting the largest changes in gene expression (Figure 3A and Supplemental Table 2). DEGs were then clustered to determine those that were coexpressed and could be defined as modules (Supplemental Table 3) (12). Gene set enrichment analysis was conducted on these modules to determine pathways associated with inflammation. Frequencies of each enriched pathway were calculated for both epithelial and immune cell types. The frequently enriched pathways within the epithelial cells included antigen processing and presentation, phagosome, intestinal immune network for IgA production, and cell adhesion molecules (CAMs). In modules from immune cell types, the frequently enriched pathways included VEGF signaling, phagosome, and NOD-like receptor signaling, though the frequencies of these pathways were lower than those in the epithelial cell modules. Of note, antigen processing and presentation, phagosome, and CAM pathways were also enriched for immune cells (Figure 3B and Supplemental Figure 2, A and B).

The most frequently enriched pathways were further explored by calculating module scores based on the genes contributing to each epithelial module pathway (Figure 3C). Module scores for CAMs and intestinal immune network for IgA production were increased in inflamed stem cells, TA cells, and immature enterocytes. In contrast, focal adhesion and ECM-receptor modules were markedly enriched in noninflamed stem and TA cells. The same approach was taken to examine the frequently enriched module pathways from immune cell types (Figure 3D). The NOD-like receptor signaling pathway module was enriched in inflamed T cell populations, and the phagosome was enriched in inflamed stem, TA, and immature enterocyte cells. In contrast, the VEGF signaling pathway was enriched in noninflamed stem, TA, immature enterocyte, and goblet cell populations. Together, focal adhesion pathway, ECM pathway, and VEGF pathway were less pronounced during active inflammation. These signatures may indicate pathways involved in downregulating inflammation or could be signs of mucosal healing in the ileum within individuals with CD.

Several HLA genes were detected across module pathways. The DEGs contributing to these pathways were assessed per cell type (Supplemental Figure 2C). Stem and TA cells had significantly increased expression of HLA-related module genes (*HLA-DPA1*, *HLA-DPB1*, and *HLA-DQB1*) during active inflammation. Additionally, BIRC3 and JUN from the focal adhesion module were significantly higher in T cells and B cells during inflammation, respectively. Taken together, these signatures point to pathways, particularly in the epithelial cells in the base of the crypts, that are undergoing changes during inflammation of the ileal mucosa.

### Global cell communication analysis reveals distinct differences in signaling networks across cell populations during inflammation in CD.

**Figure 4:**
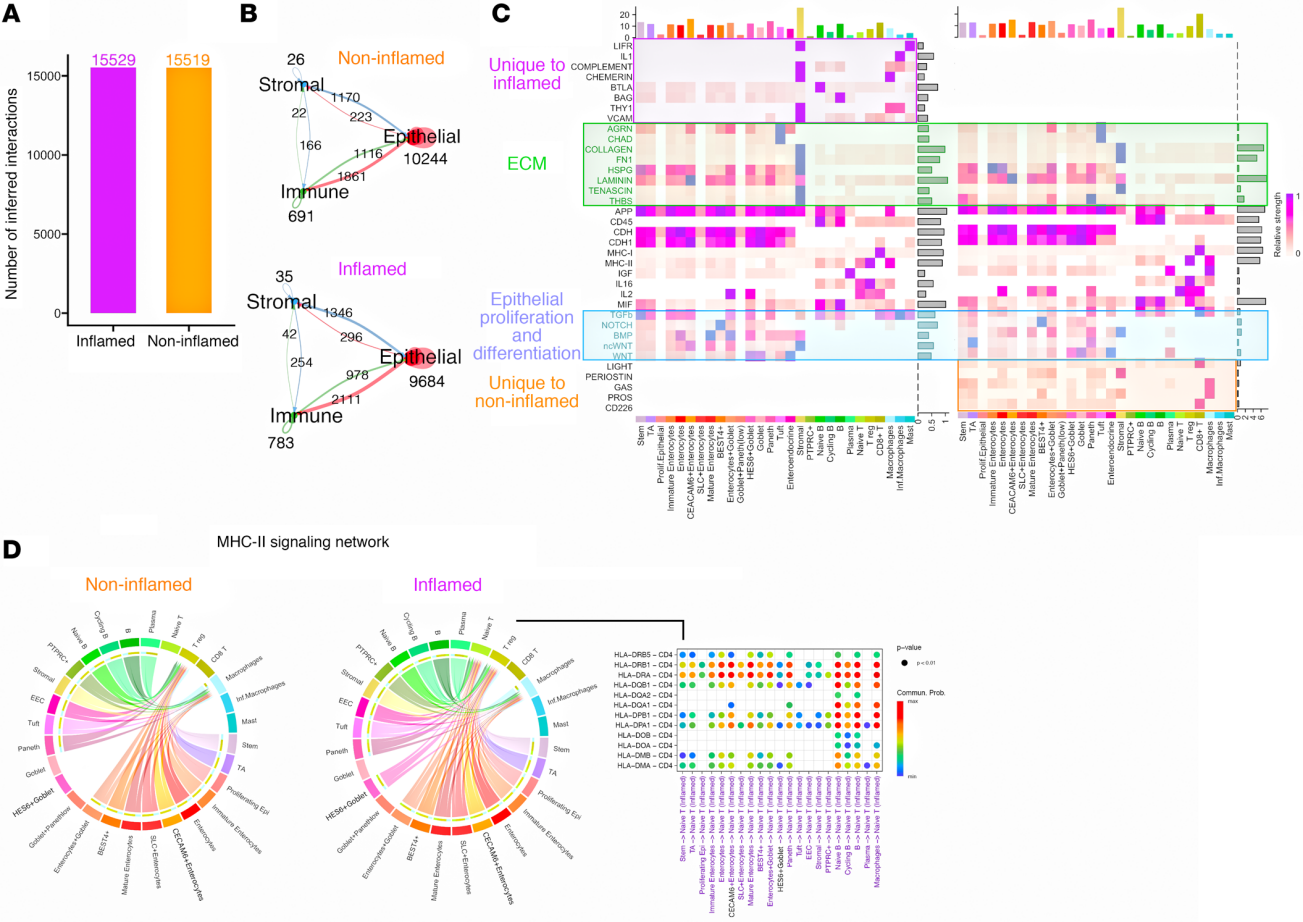
Altered putative cell signaling networks in inflamed compared with noninflamed CD. (**A**) Bar plot of total number of possible RL interactions. (**B**) Number of RL interactions between the 3 major compartments. (**C**) Heatmap illustrating the selected signaling patterns in inflamed and noninflamed CD. (**D**) Chord diagrams depicting the MHC class II (MHC-II) signaling network (left 2 plots) and dot plot showing RL signaling toward naive T cells uniquely in inflamed (right). Edge color in the chord plot represents the signaling source, and segments with arrows are the signaling targets.

Given the changes in antigen processing and presentation and cell adhesion activity during inflammation, we next investigated the intercellular communication differences between inflamed and noninflamed CD samples. Using CellChat, we analyzed interactions among 28 cell populations in inflamed and noninflamed groups (Supplemental Table 4), as well as in individual CD patient samples (Supplemental Figure 3), to better understand the patient heterogeneity. We examined the total number of incoming and outgoing interactions and cellular compartments most perturbed by inflammation. Our initial analysis showed a similar total number of interactions in both groups (more than 15,000 RL pairs) (Figure 4A). However, at the cellular-compartment (epithelium, immune, and stromal) level, the inflamed mucosa exhibited a loss of 560 predicted epithelial interactions, alongside an increase of 92 immune and 9 stromal interactions, compared with noninflamed. Notably, the interactions of stromal cells to both immune and epithelium, followed by epithelium to immune, were predicted to be stronger within the inflamed CD mucosa (Figure 4B).

We then examined pathway patterns by calculating each cell type’s incoming and outgoing signal strength. This analysis revealed several pathways that were unique to inflamed ileal CD, including interleukin-1 (*IL1*), vascular cell adhesion protein (VCAM), and *THY1* membrane glycoprotein. In contrast, pathways like protein S, *CD226*, and growth arrest-specific protein networks were uniquely enriched in noninflamed ileal mucosa. Notably, pathways linked to epithelial proliferation and differentiation, such as BMP, NOTCH, and WNT, were enriched in noninflamed CD, while ncWNT was upregulated in inflamed CD. We also observed increased signaling of key inflammatory pathways, including APP and MHC class II (MHC-II), in inflamed CD. Additionally, pathways involved in ECM, including AGRN, FN1, CHAD, and HSPG, were upregulated in inflamed CD (Figure 4C and Supplemental Figure 4).

Next, we assessed the flow of information among cell populations involved in the MHC-II (antigen presentation) pathway based on communication probability. In inflamed CD, the chord diagram shows naive T cells as the signaling target and early epithelial cells as the signaling source, a pattern absent in noninflamed tissue. Additionally, the RL pairs mediating this signaling toward naive T cells were elevated across several cell types in inflamed CD (Figure 4D).

### Inflamed Crohn’s ileal mucosa indicates dynamic alterations in intercellular signaling.

**Figure 5:**
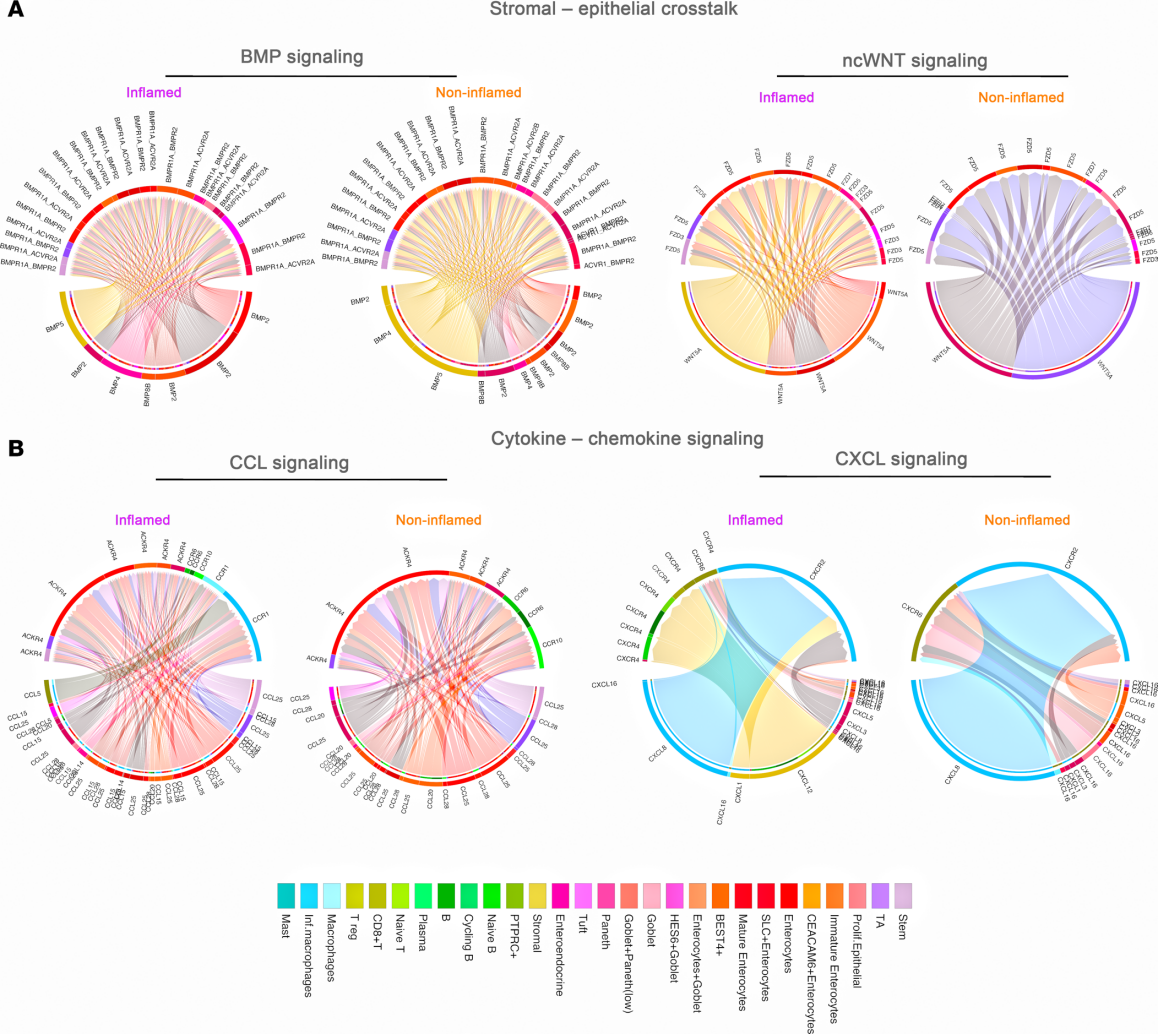
Dynamic alterations in signaling pathways in the inflamed Crohn’s ileal mucosa. (**A**) Inferred BMP and ncWNT RL communications between stromal and epithelial cell types. BMP, bone morphogenetic protein; nc, noncanonical. (**B**) Inferred CCL and CXCL RL pairs across multiple cell types.

We then investigated the signaling mechanisms that were potentially altered during inflammation. We identified dysregulated RL pairs involved in epithelial differentiation pathways during inflammation. We detected changes in BMP and ncWNT signaling, pathways crucial for maintaining intestinal crypts. We also found that the source of BMP2 and BMP4 ligands shifted from stromal cells and epithelial subtypes in noninflamed mucosa to primarily epithelial subtypes in the inflamed. Additionally, the WNT5A ligand in the noncanonical WNT pathway, expressed by Paneth and TA cells in noninflamed mucosa, was primarily expressed by enterocyte subtypes and stromal cells during inflammation (Figure 5A).

Chemokines essential for wound healing were predicted to be altered during inflammation. In the CCL network, macrophages and inflammatory macrophages emerged as signaling targets through the *CCR1* receptor in the inflamed mucosa. Likewise, in the CXCL pathway, B cell subpopulations, naive T cells, Tregs, and macrophages became signaling targets in the inflamed mucosa. This contrasts with the noninflamed mucosa, where macrophages and CD8^+^ T cells were the cellular targets during inflammation (Figure 5B). Together, our findings predict dynamic alterations in signaling strength and RL communication architecture during inflammation.

### Non-negative matrix factorization demonstrates spatial compartmentalization and localizes active pathways represented in CD.

**Figure 6:**
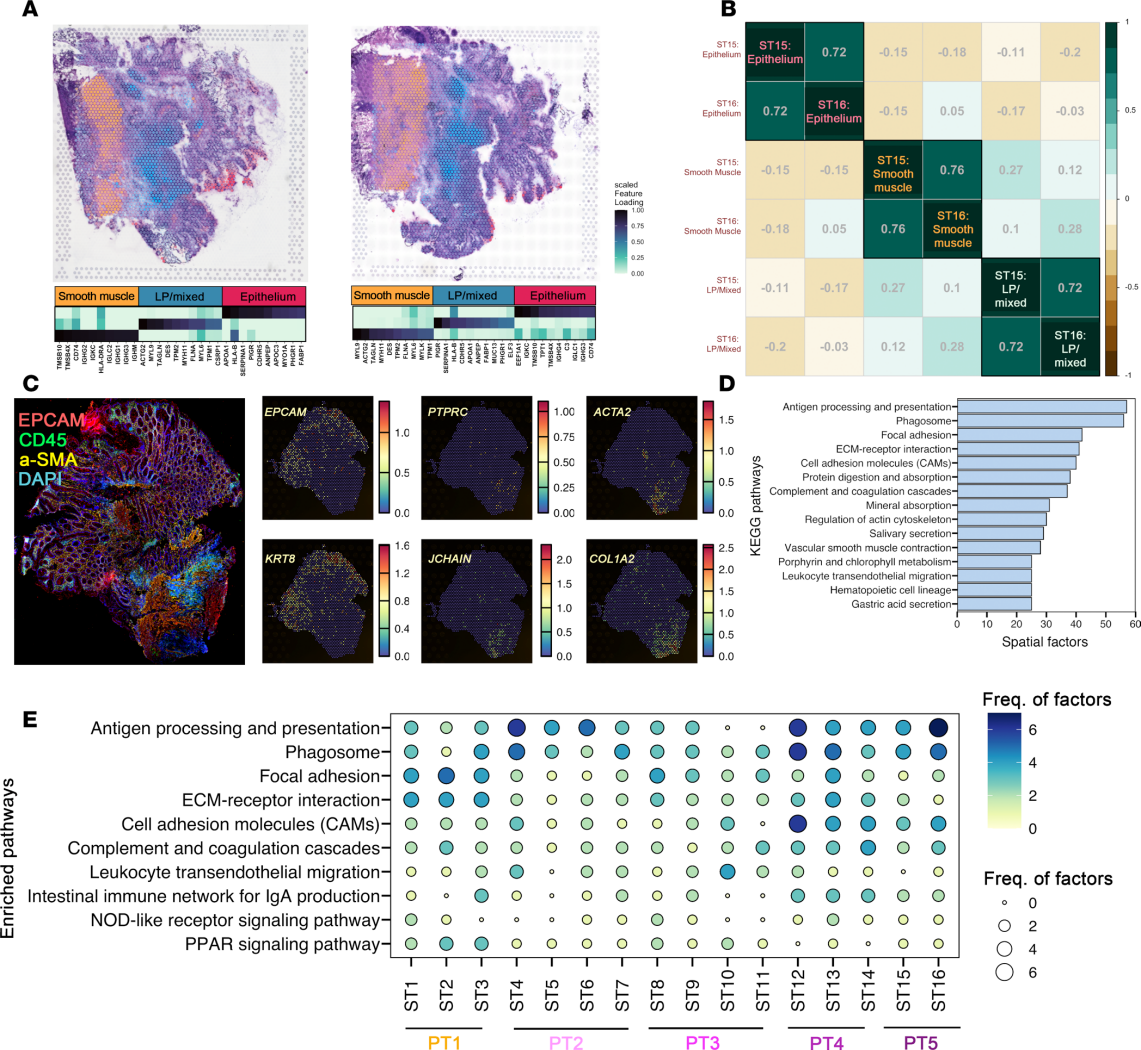
Non-negative matrix factorization delineates compartmentalization and regionalization of disease-implicated pathways. (**A**) Mapping of non-negative matrix factorization (NNMF) (*n* = 3 factors) genes onto representative spatial tissues ST15 and ST16 from patient 5. LP, lamina propria. (**B**) Correlation of factors across spatial tissue sections from patient 5. (**C**) Immunofluorescence (left) and RNA expression (right) of markers corresponding to compartments. a-SMA, α–smooth muscle actin. (**D**) Top pathways enriched across all patients using NNMF (*n* = 10 factors per slide). (**E**) Dot plot representing enriched pathways corresponding to factors per patient tissue.

Next, we conducted ST on 16 surgically resected ileal tissues from 4 inflamed and 1 noninflamed CD patients (Table 2 and Supplemental Figure 5) that were all processed using the 10x Genomics Visium platform. We first explored the basic transcriptomic patterns underlying each tissue using 3 factors generated by non-negative matrix factorization (NNMF) (13). The 3 factors distinguished the epithelium, lamina propria/mixed, and smooth muscle compartments (Figure 6A and Supplemental Figure 6) (13). Factors were compared across the same patient’s tissue sections obtained serially. Annotated factors were highly correlated and offered evidence of similar transcriptional activity being retained in tissues from the same patient (Figure 6B and Supplemental Table 5). This correspondence suggests that the ST data provide robust representation of patient mucosal biology. The labeled compartments were also verified by antibody staining using immunofluorescence and transcriptomic expression of canonical cell markers (Figure 6C). To uncover more distinctive, regionalized activity that was sufficiently represented across the slides, we conducted pathway enrichment analysis on 10 detailed factors per slide and assessed those that were most frequently enriched (Figure 6D). Frequently enriched pathways included antigen processing and presentation, phagosome, ECM-receptor interactions, CAMs, and focal adhesions that were also consistently observed when examining pathways per patient (Figure 6E).

### Combinatorial single-cell and spatial transcriptomics depict heterogeneous mucosal microdomains of inflammatory activity.

**Figure 7:**
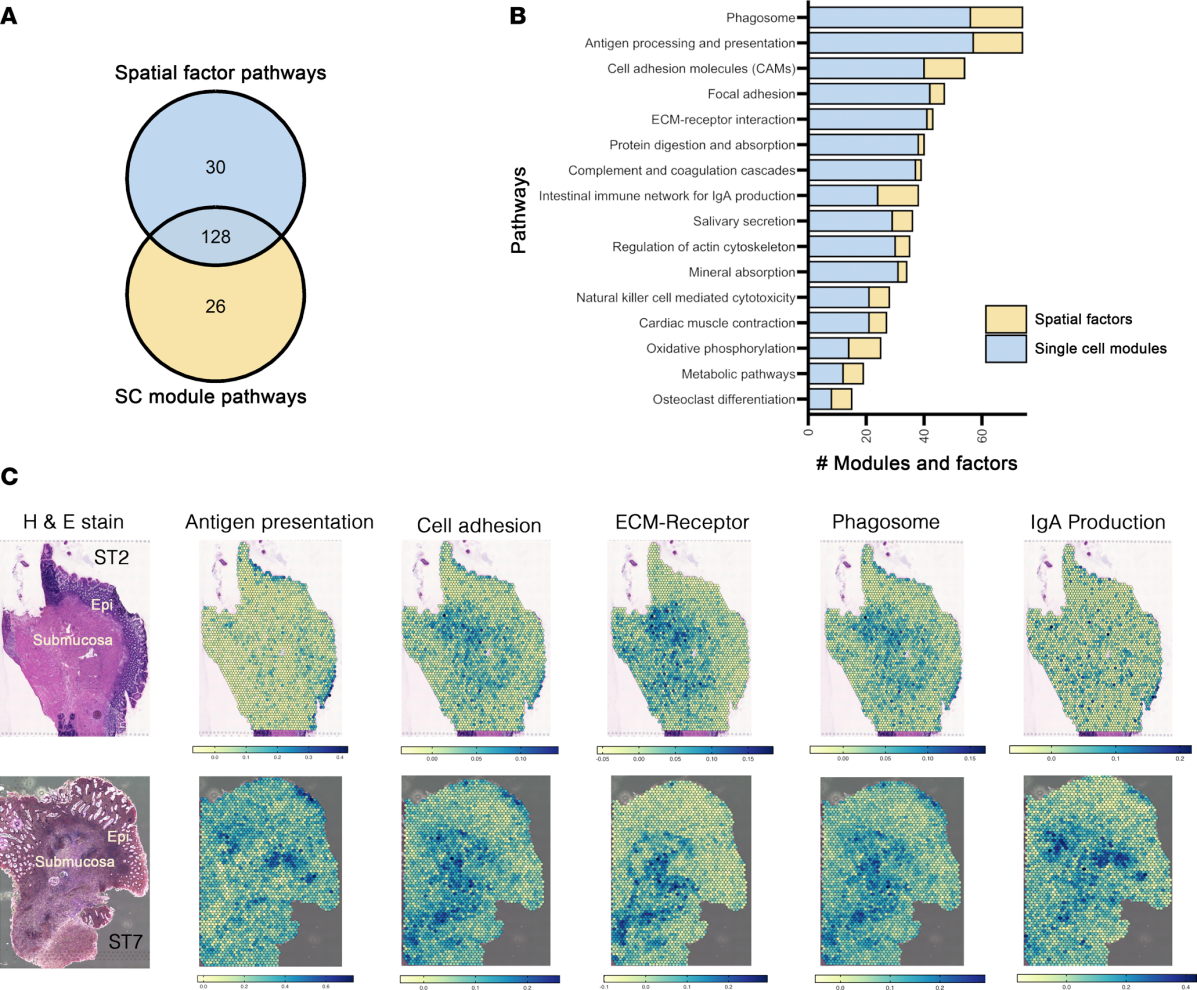
Combinatorial single-cell and spatial transcriptomics depict heterogeneous mucosal microdomains of inflammatory activity. (**A**) Venn diagram of numbers of intersecting and unique enriched pathways between ST factors (top blue circle, “Spatial Factor Pathways”) and single-cell transcriptomic modules (bottom yellow circle, “SC Module Pathways”). (**B**) Frequencies of top KEGG pathways enriched in both spatial factors and single-cell modules. (**C**) Mapping cumulative expression of pathway genes from combined methods onto noninflamed ST2 from patient 1 (top) and inflamed ST7 from patient 2 (bottom) spatial tissue sections.

To gain more spatial context into the mucosal architectural changes taking place during inflammation, we leveraged the gene modules and RL interactions from our parallel single-cell and ST analyses and examined the intersecting and unique pathways across the 3 methods. Of the 184 biological pathways observed across spatial and single-cell analysis, 128 (~70%) were detected in both, while 30 were unique to spatial analysis and 26 unique to single-cell analysis (Figure 7A). Among the overlapping pathways were antigen processing and presentation, phagosome, CAMs, intestinal network for IgA production, and ECM-receptor signaling (Figure 7B, Supplemental Figure 7, and Supplemental Table 6). These pathways were among the most frequently enriched in the individual analyses and point to pathways most affected by chronic inflammation in CD. To examine these biologically relevant findings in their spatial context, we first sought to define an integrative gene list for each pathway. Gene lists were curated for each pathway by extracting the genes that contributed to pathways from modules, RL pairs, or factors (Supplemental Table 7). Gene lists for the antigen processing and presentation, phagosome, and CAM pathways included several HLA genes, while gene lists for focal adhesion and ECM-receptor pathways included integrins and additional ECM-related genes.

Identifying genes from these pathways represented across these methods revealed specific mapping on spatial tissues. Based on localization, we observed specific and high enrichment of antigen processing and presentation in the epithelium for both inflamed and noninflamed ST tissue with increased enrichment into submucosa within inflamed tissue as shown in a representative sample in Figure 7C (Supplemental Tables 5 and 8). Phagosome, CAMs, ECM-receptor interactions, and intestinal network for IgA production pathways were localized within the submucosa and epithelium in both noninflamed and inflamed tissue. However, enrichment was less distributed and more focused in patches within the inflamed mucosa. Phagosome, CAMs, and ECM pathways colocalized with one another in the inflamed submucosa, whereas antigen presentation and IgA production activity were more colocalized than with the other pathways. This evidence conveys that disease activity is not uniformly distributed but occurs in pockets of inflammatory microdomains localized deep in the mucosa and within the epithelial layers during active CD.

### Conditional AutoRegressive-based Deconvolution on ST tissues suggests macrophage infiltration within regions highly enriched in epithelial antigen presentation and CD74 expression in inflamed CD mucosa.

**Figure 8:**
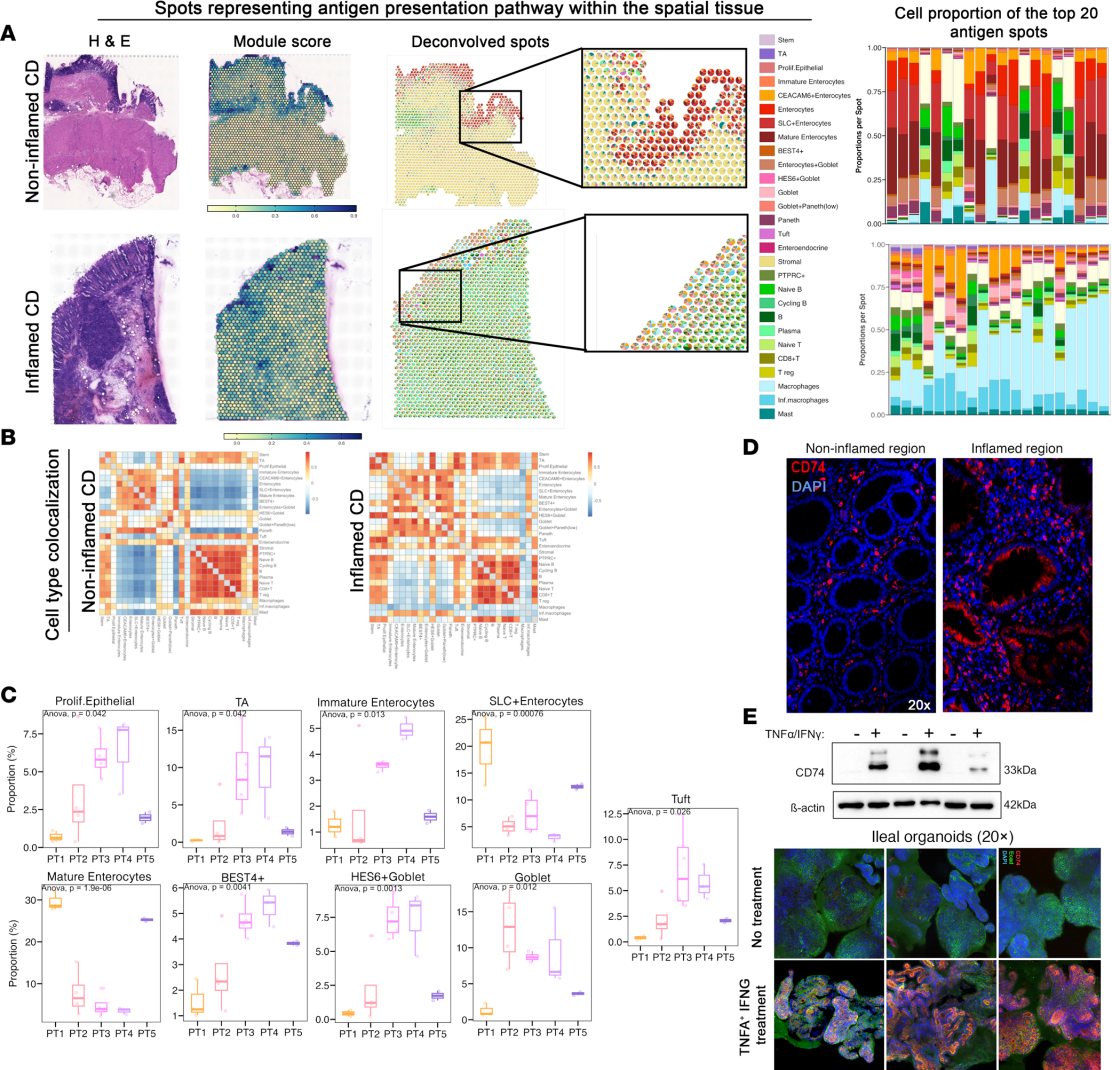
CARD on ST tissues within regions highly enriched in antigen presentation. (**A**) For spatial tissues ST1 (patient 1, noninflamed) on top and ST14 (patient 4, inflamed) on bottom. Plots from left to right represent hematoxylin and eosin staining (leftmost panel), overlay of antigen processing and presentation genes onto spatial tissues (second to leftmost, color coded with yellow representing low score and blue as high score), cell type deconvolution of spatial tissues with expanded view of regions containing high enrichment of antigen processing and presentation genes as pies (second to rightmost), and stacked bar plot of 20 representative spots’ cell type proportions (rightmost). (**B**) Cell type colocalization corresponding to 20 representative highly enriched antigen processing and presentation spots in ST1 (patient 1, noninflamed) on left and ST14 (patient 4, inflamed) on right. (**C**) Proportional changes in highly enriched antigen processing and presentation spots per slide per cell type (*y* axis) compared across patients (*x* axis). (**D**) Immunofluorescence staining of CD74 (red) with DAPI (blue) in the ileal mucosal epithelium in noninflamed (left) and inflamed (right) CD (*n* = 4). (**E**) Western blot of CD74 using β-actin as loading control (left) in nontreated (NT) and IFNG/TNFA-treated ileal organoids and immunofluorescence (right) of CD74 (red) and E-cadherin (Ecad) (green) with DAPI (blue) of ileal organoids without and with IFNG/TNFA treatment (*n* = 3).

Finally, we investigated if we could gain a higher resolution picture of the cellular milieu in the inflammatory microdomains by leveraging Conditional AutoRegressive-based Deconvolution (CARD) (15) to deconvolute cell types within 20 representative spots highly enriched in the antigen presentation pathway (Figure 8A and Supplemental Figure 8). Deconvolved spots in the noninflamed CD mucosa depicted a larger repertoire of epithelial subtypes, including more mature enterocytes, and comprised immune subtypes, such as Tregs, CD8^+^ T cells, and mast cells, that were less abundant in the inflamed CD mucosa. In fact, mast cells appeared more focused in the noninflamed mucosa, whereas they were more distributed across all the spots in the inflamed mucosa. Inflamed mucosa consisted of more immature epithelial cell types (stem, TA, proliferating epithelial, and *HES6*^+^ goblet cells) colocalizing with multiple immune cell populations, verifying that antigen presentation from epithelial cells to immune cells may predominantly occur at the base of the crypt. Within the inflamed mucosal tissue, macrophages were one of the most abundant cell types colocalizing within the epithelial domains. Such features could not be readily discerned in the noninflamed CD surgical tissue section (Figure 8A). Migration of activated macrophages into the inflamed epithelium suggests CAM involvement and is also observed at the protein level (Supplemental Figure 9). Figure 8B conveys a heatmap representation of cell types observed together within the spots and their likelihood of colocalization. Among global changes in cell type composition within regions with active antigen presentation in noninflamed and inflamed CD tissues, it is noteworthy that interactions with stromal cells are diminished across many cell types where antigen presentation is taking place. Increases in *PTPRC*^+^ cells colocalizing with epithelial subtypes were also apparent in inflamed tissue regions (Figure 8B). In contrast, the noninflamed CD mucosa revealed more stromal cells within these regions colocalizing with multiple epithelial and immune subtypes. Cellular proportion differences across ST tissue slides analyzed per patient further support increased early epithelial cell types, reduced mature enterocytes, and increased goblet proportions, particularly early epithelial cells, in regions of active antigen processing and presentation during inflammatory CD (Figure 8C). Using immunofluorescence on FFPE ileal tissue sections, we tested for the presence of antigen presenting pathways with CD74 (Supplemental Table 7) as a marker (16, 17). We observed increased expression of CD74 in epithelial cells in the inflamed mucosa (Figure 8D). We tested using ileal organoids whether inflammatory mediators were increasing CD74 expression levels within epithelial cells by adding TNFA/IFNG to the culture medium. At 48 hours following the addition of the cytokines, CD74 was readily detectable by Western blot and immunofluorescence but undetectable in the untreated organoids (Figure 8E).

## Discussion

The purpose of this study was to gain insights into the molecular cell biology of mucosal inflammation during ileal CD using a combinatorial analysis of single-cell and spatial transcriptomics. We identified significantly enriched pathways across our single-cell gene modules, RL interactions, and spatial NNMF that point to inflammation-driven changes in antigen presentation, cell adhesion, phagosome, intestinal immune network for IgA production, focal adhesions, and ECM signaling. We then localized the regional distribution for these biological processes in the spatial tissues and identified the cell subtypes involved.

Antigen processing and presentation has been shown to be associated with inflammation and CD through several genomic and transcriptomic studies (18–20). As expected, this pathway consisted of several HLA genes from our combinatorial analysis and was enriched in specific regions comprising epithelial and immune cells within our spatial tissues. Multiple HLA alleles have been implicated from high-density SNP assessments in both CD and ulcerative colitis (21). The specific localization of HLA genes to the tissue epithelium is aligned with previous studies implicating the role of epithelial subtypes as nonconventional antigen-presenting cells in CD. In particular, the enrichment of antigen activity in the epithelium may suggest crosstalk between the epithelial and immune compartments, as the canonical interaction of MHC-II antigens with T cells has been shown to modulate epithelial cell fate and barrier maintenance (18, 22). To this end, our single-cell analysis identified increased HLA gene expression (HLA-DP, HLA-DQ, HLA-DR, HLA-DM) in undifferentiated epithelial cell types, including stem and TA cells, and MHC-II signaling (HLA-DP, HLA-DQ, HLA-DR, HLA-DM, HLA-DO) coming uniquely from *HES6*^+^ goblet cells (23) and to naive T cells during active inflammation. Our spatial findings using CARD-deconvolved cell type proportions verified the increased composition of early epithelial cells and other crypt base cells in spots exhibiting high antigen presentation expression within the inflamed tissues.

We additionally verified the expression of *CD74* associated with antigen processing and presentation across our analysis methodologies. Previous studies have noted that *CD74* protein is expressed in the mucosal epithelium during CD but is relatively absent in non-IBD controls (7, 16). From our study, we demonstrate that there also exists a difference in *CD74* expression between noninflamed and inflamed CD. We extended these findings in multiple epithelial cell types at the RNA level as well as at the protein level within the inflamed epithelium. From our combined single-cell and ST analysis, we observed enriched communications between *CD74* and multiple genes, including *CXCR4*, *CD44*, *MIF*, and *APP*, which were among the antigen processing molecules regionalized in the tissue epithelium.

Our data also demonstrate altered epithelial-immune interactions corresponding to communication with macrophage populations. We found increased proportions of inflammatory macrophages in the vicinity of crypt bottom epithelial cells (Stem, TA, Prolif.Epithelial, and HES6+ goblet) residing within the inflamed tissue section. In contrast, these patches of microdomains within noninflamed CD tissues exhibited high composition of mature enterocytes with minimal proportions of macrophages. Garrido-Trigo et al. employed single-cell transcriptomics and CosMx spatial molecular imaging and detected higher proportions of inflammation-dependent alternative macrophages in the mucosal epithelium in patients with IBD (24). They found these cells to be overexpressing neuregulin-1, which is suggested to play a role in epithelial regeneration.

Chemokines, cytokines, and CAMs are crucial modulators in the wound-healing processes by mediating the recruitment of inflammatory cell types (25, 26). Our single-cell analysis highlights these distinct putative molecules and their signaling networks that vary across inflammatory states of CD. Within the inflamed CD subset, we identified a unique enrichment of pro-inflammatory *CCL5* produced by CD8^+^ T cells and an emerging VCAM signaling network alongside epithelial production of chemokines (27). These signals induce a microenvironment that recruits macrophages and other leukocytes to the site of inflammation. One evidence substantiating this is signaling to *CCR1* receptor on macrophages and inflammatory macrophages exclusively in inflamed CD. These recruited macrophages and inflammatory macrophages also express *CXCL8* and *CXCL16* ligands that might further contribute to the induced inflammation. In addition to macrophages, stromal cells in the inflamed CD produce *CXCL12*, a lymphocyte recruitment factor secreted in response to epithelial damage (28). Supporting this, we also found significant decreases (Figure 2D) in epithelial cell proportions, including enterocyte subtypes and enteroendocrine cells, during inflammation. In summary, while chemokines and cytokines play an important role in wound healing at early stages, a chronic inflammatory response aggravated by these signaling pathways can lead to failure in tissue repair.

Apart from influencing intestinal immune cells, stromal cells are key regulators of ECM. ECM components are primarily secreted from the mesenchyme and involve factors from the other compartments. Inflamed CD presented distinct communications originating from stromal and epithelial subtypes across all ECM pathways. In collagen and laminin pathways, we observed increased signaling from stromal to CD8^+^ T cells. Collagen and laminin genes also play a role in the cell adhesion molecule pathway, which is essential for barrier maintenance. Superimposing CAM pathway genes onto the inflamed spatial tissues revealed greater proximity between stromal and immune cells, including CD8^+^ T cells (29).

Stromal cells also play a central role in regulating intestinal epithelial cell renewal (30). Although we observed only a predicted moderate increase in stromal-epithelial communication within the inflamed phenotype, we identified a predicted decrease in BMP signaling from stromal cells in patients with active CD. These stromal-epithelial interactions may contribute to the differences in epithelial cell composition observed between CD phenotypes.

There were a few limitations to our study. Our analysis included relatively small sample sizes, and all 3 noninflamed spatial tissues were from the same patient. Although we delved into signatures represented in both single-cell and spatial transcriptomics, we also detected features that were not replicated across the methodologies. These may be attributed to differences in disease time points and resolution. We also recognize that CD is a heterogeneous disease, and the differences we observe may represent biological variations corresponding to each patient. Future experiments will expand on the sources of these signals using additional time points, samples, and impacted regions beyond the ileum. Together, our multiomics study provides a deeper resolution into coordinated molecules and their 2D positioning from tissue sections spanning across severities of CD pathogenesis.

## Methods

### Sex as a biological variable.

Our study includes male and female patients with CD, but sex was not considered as a biological variable.

### Patient specimen collection.

All patients participating in this study were diagnosed with CD, but their disease varied in severity. Ileal tissue and mucosal biopsies from patients with CD undergoing surgery or clinically indicated colonoscopy at Children’s Healthcare of Atlanta (CHOA) were used in this study under approved IRB protocols. Patient demographics for samples that underwent single-cell RNA sequencing are shown in Table 1. Endoscopic and histological activity scores were evaluated for all 15 patients. Eight biopsies were macro- and microscopically inflamed, and 7 were macroscopically (endoscopically) inflamed but determined by pathology to be microscopically noninflamed. Tissue sections obtained from different regions of ileal surgical specimens were processed for ST, as phenotypically detailed in Table 2; *n* = 5 patients, 16 sections total. The inflammation status of each section of surgical tissue (microscopically inflamed or noninflamed) was assessed by a pathologist at CHOA.

### Single-cell transcriptomics processing.

Biopsies were processed immediately after endoscopy using a cold-protease (MilliporeSigma catalog P5430) protocol. Biopsies were minced on ice with microscissors in the presence of protease in Dulbecco’s PBS with 2.5 mM EDTA, Rho kinase inhibitor (Y-27632) (catalog ab120129), and caspase inhibitor (catalog ab141421) obtained from Abcam. Samples were kept on ice for 15 minutes and vortexed intermittently. After 40 μm filtration, the filtrate was protease-digested on ice for an additional 15 minutes and then filtered through the same 40 μm filter (Thermo Fisher Scientific) Recovered cells were washed and then subjected to a 20 μm filtration step (pluriSelect filter). A target of 7,000–10,000 encapsulated cells were loaded into the 10x Genomics Chromium Controller following the manufacturer’s protocol with the 10x Genomics 3′ single-cell RNA-sequencing kit v3.1 (Product code: 1000268). The cDNA and libraries were prepared following 10x Genomics workflows and sequenced on an Illumina NovaSeq S4 kit sequencing platform at 50,000 reads per cell.

### ST processing.

Surgical resections of CD mucosa (B1, B2, and B3 disease) were flash-frozen in optimal cutting temperature compound using an ultracold isopentane/liquid nitrogen bath, cut into 10 μm tissue sections, and processed for ST according to manufacturer’s protocol as follows. The sections were placed on Gene Expression slides (PN-100188) within the fiducials harboring 5,000 barcoded capturing spots. Sections were fixed in ice-cold methanol and stained with H&E or processed for immunofluorescence using antibodies for the epithelial marker *EPCAM* obtained from MilliporeSigma (catalog MAB26); the immune cell marker *CD45* from Cell Signaling Technology (*CD45/PTPRC*) (catalog AB13917); and the stromal marker, α–smooth muscle actin, obtained from Dako (catalog MO851), and nuclear stain DAPI from Thermo Fisher Scientific (catalog 62248). Images were captured on a Keyence BZ-X800 microscope. Optimization of the permeabilization step for RNA capture using the 10x Genomics Visium Optimization Kit (PN-100191) resulted in an 18-minute permeabilization time. After permeabilization, polyadenylated RNAs were reverse-transcribed and barcoded, and then the cDNAs were used for library construction, all using the Visium Gene Expression Kit (PN-1000185). Libraries were sequenced at a read depth of 6,000 reads per spot. The software 10x Genomics Space Ranger v1.0 was used to align and count unique molecular identifiers against 10x Genomics GRCh38 human reference genome for each Visium ST spot.

### Single-cell transcriptomics processing, quality control, and clustering analysis.

FASTQ files were aligned to the GRCh38 human genome using Cell Ranger 6.1.0 or 7.1.0 (31). We integrated uncorrected gene expression matrices generated per sample from both inflamed (*n* = 8) and noninflamed (*n* = 7) CD phenotype, revealing clustering based on the features per cell, ambient RNA expression, and mitochondrial percentage. These differences often hinder genuine biological signatures. Consequently, we performed careful downstream analysis to mitigate technical artifacts. We removed counts due to random barcode swapping and ambient RNA molecules from raw Cell Ranger outputs using the remove-background module in the CellBender software (32). Subsequently, from each CellBender matrix, using Seurat R package (v5.0.1) (33), we pruned both mitochondrial and ribosomal genes (to remove their effect on clustering), identified and removed low-quality cells or empty droplets (features per cell ≤ 100) and cells with > 80% mitochondrial proportions, and applied DoubletFinder to remove potential doublets (34). The resulting filtered gene expression matrices underwent log normalization by a scaling factor of 10,000 and dimensionality reduction using principal component analysis (PCA) using 30 PCs. We applied reciprocal PCA batch correction to the resulting matrices to remove technical variation. We computed local neighborhoods and applied clustering with a resolution of 0.5 based on 30-dimensional PC space. To visualize, we used UMAP embedding to capture the structure in the dataset. We identified the top 20 marker genes per cluster by performing the Wilcoxon rank sum test using the FindAllMarkers function with gene expression in at least 25% of the cells and log fold-change value of at least 0.25. We annotated each cluster based on gene expression of previously published canonical cell type markers (6, 8, 35–37) (Figure 1 and Supplemental Table 1) and cell cycle phases.

### Single-cell trajectory analysis.

To infer this dataset’s epithelial and immune trajectories, we computed a neighborhood graph from a 30-dimensional PCA space within the 2 compartments separately. PAGA was performed on the individual neighborhood graphs to estimate the connectivity between the cell types (38).

### Cell-cell communication using CellChat.

We employed CellChat to analyze the cell-cell communication between the cell types in inflamed and noninflamed CD phenotypes (11). For the analysis, we created a CellChat object based on cell type groupings and used the “CellChatDB.human” ligand-receptor interaction database. All the preprocessing steps were implemented with default parameters. CellChat functions *computeCommunProb* and *computeCommunProbPathway* were used to infer the RL pairs and signaling pathways.

### Single-cell inflammation-associated genes.

To validate our histological assessment of inflammation, we created gene signature scores of the inflammation-associated genes (8): *IFNG*, *IFNGR1*, *IFNGR2*, *IL10*, *IL12A*, *IL12B*, *IL12RB1*, *IL12RB2*, *IL13*, *IL17A*, *IL17F*, *IL18*, *IL18R1*, *IL18RAP*, *IL1A*, *IL1B*, *IL2*, *IL21*, *IL21R*, *IL22*, *IL23A*, *IL23R*, *IL2RG*, *IL4*, *IL4R*, *IL5*, *IL6*, *JUN*, *NFKB1*, *RELA*, *RORA*, *RORC*, *S100A8*, *S100A9*, *STAT1*, *STAT3*, *STAT4*, *STAT6*, *TGFB1*, *TGFB2*, *TGFB3*, and *TNF*. We calculated the inflammation score using AddModuleScore and then combined these values by calculating the mean per sample. The *P* values (*P* < 0.05) between noninflamed and inflamed were obtained using Wilcoxon’s test per cell type.

### Single-cell cell type proportion and differential gene expression analysis.

Proportions of each cell type were compared in the inflamed and noninflamed groups and visualized in box plots. Differential gene expression analysis was performed for each cellular subtype with the Seurat (v5.0.1) (33) FindMarkers() function using the MAST algorithm, with the inflamed category as the first group and the noninflamed category as the second group (39). All DEGs in each cell type are in Supplemental Table 2. Gene set enrichment analysis was performed separately for the upregulated and downregulated genes using the clusterProfiler R package (v4.8.3) and the KEGG database (updated March 1, 2024) (40, 41).

### Single-cell gene module analysis.

For each cell type, DEGs were filtered for significance (adj. *P* < 0.05), and unsupervised hierarchical clustering was performed on the absolute value of genewise log_2_ fold-changes using the average of the distances between points in each cluster as the overall distance between clusters in the hclust R function (12). The clustering results were cut into at most 20 modules for each cell type using the cutree function (all clustering results are in Supplemental Table 3), and gene set enrichment analysis was performed using clusterProfiler (v4.8.3) and the KEGG database for each gene module (40, 41). The overall frequencies of enriched pathways were calculated for epithelial and immune cell types separately; to facilitate the identification of biologically relevant processes, disease- and infection-related pathways were also filtered out (Figure 2B), with the full pathway lists included in Supplemental Figure 2A. Module scores of modules of interest were plotted in heatmaps using the ComplexHeatmap package (v2.16.0) (42).

### ST quality control and processing.

ST data for 16 slides from 5 patients (Table 2) were normalized using scTransform (Seurat v5.0.3) and quality filtered by excluding the mitochondrial and ribosomal genes from the transcripts detected per spot (33). Distributions of quality metrics are included in Supplemental Figure 5. These objects were then used for NNMF and pathway analysis.

### NNMF for ST.

ST data per slide were first evaluated according to their unsupervised gene expression patterns using NNMF as described by the semla package (v1.1.6) (13). Three factors per slide from NNMF were compared within patient tissue sections using Spearman’s correlation and visualized with corrplot (v0.92). Active pathways corresponding to the KEGG database were further deduced by using 10 factors per spatial slide, calculating their enrichment using enrichR (v3.2) and clusterProfiler (v4.8.3), and filtering based on their *P* significance (40, 41, 43). Frequencies of significant pathways across slides per patient and overall helped suggest pathways where we had the data available for further investigation (Supplemental Table 5).

### Integrative single-cell and ST gene set curation and analysis.

Gene lists were compiled based on overlapping pathways (Supplemental Table 6) identified from gene modules and RL interactions in single-cell data and NNMF from ST analysis, as described in Supplemental Table 7. These genes were projected onto each spatial slide to quantify their cumulative expression and localization, before deconvolving estimated cell proportions.

### CARD for cell type proportions per spot.

Cell type deconvolution per spot was conducted on all spots in spatial tissue slides based on their gene expression patterns using CARD v1.0 (15). The single-cell RNA-sequencing cell type–specific gene expression data as well as spatial localization and transcriptomics data were implemented together as input for CARD’s NNMF-based deconvolution to determine composition and relative proportions of cell types, further assessing based on spots with high expression of genes involved in explored pathways. Colocalization of cell types in the top 20 spots with high expression of these pathway genes was visualized using pheatmap (v.1.0.12). Significant proportional differences of top spots expressing pathway genes within each section were compared across patients using ANOVA and Student’s *t* tests (Supplemental Table 8).

### Organoid culture.

Patient-derived ileal organoids were grown as described previously (17) with minor modifications to the media, propagating this time in Human Intesticult Organoid media from STEMCELL Technologies. Organoids were used between 3 and 5 passages for experiments and treated with or without TNFA (catalog 210-TA-020) and IFNG (catalog 285-IF-100) obtained from R&D Systems, Bio-Techne (both at 20 ng/mL), for 48 hours.

### Western blot.

Total proteins were extracted from patient-derived organoids using 1× SDS sample buffer with 20 mM DTT. Equal amounts of protein were separated by SDS-PAGE, transferred, and probed with CD74 (catalog 77274S) and loading control β-actin (catalog A5441) from MilliporeSigma. Secondary antibodies were obtained from NA934V and NA931V from GE Healthcare (now Cytiva).

### Immunofluorescence.

Organoids were fixed in 4% formaldehyde/PBS, permeabilized with 0.5% Triton X-100, and blocked with 5% BSA/PBS before antibody detection. *CD74* was detected with mouse anti-CD74 (Cell Signaling Technologies catalog 77274) from Abcam, and Ecad (catalog 14472) from Cell Signaling Technology was detected with mouse anti-rabbit. Antibodies were detected with anti-mouse and anti-rabbit Alexa Fluor 488 (catalog A11001) and 555 (catalog A32732) from Thermo Fisher Scientific, respectively, and the nuclei were stained with DAPI. For immunofluorescence in ileal CD tissue sections, FFPE ileal tissues were used. After deparaffinization, tissues were incubated with CD74 antibodies (Figure 8D). For the Multiplex assay, we used Lunapore COMET to stain CD68, CD11c, Ecad, and CD74 antibody (Supplemental Figure 9C) along with 16 other antibodies (data not shown).

### Statistics.

Statistical tests were used to evaluate significant differences between the inflammatory groups in CD. Wilcoxon’s test and 2-tailed Student’s *t* test were used to evaluate significant inflammatory score and proportional differences, respectively, between inflamed and noninflamed CD groups using *P* < 0.05. Pathway enrichment with *P* < 0.05 was determined by significant DEGs with *P* < 0.05 and log_2_ fold-change > 0.25 using MAST algorithm.

### Study approval.

The Institutional Review Board of CHOA approved the study, and written informed consent was obtained from all participants or their guardians.

### Data availability.

The dataset used to determine the conclusions from this article is available at NCBI Gene Expression Omnibus under the accession ID GSE228360. The data supporting the results are provided in the Supporting Data Values file. The code used for the single-cell and ST data analysis is available at https://github.com/SubraLab/Spatial-IlealCD (commit ID a88cad1).

## Author contributions

Conceptualization was done by SK, JDM, and VLK; funding was acquired by SK, PQ; investigation was done by AFD, VLK, JDM, GS, NJ, DG, RSP, PQ, MA, GNJ, S Murthy, SCM, YH, SV, TK, DJC, HY, CAL, and S Munasinghe; formal analysis was done by YH, S Murthy, and SCM; visualization was done by YH, S Murthy, SCM, and VLK; writing of the original draft was done by VLK, S Murthy, SCM, YH, and JDM; and review and editing were done by JDM, VLK, and SK.

## Supplementary Material

Supplemental data

Unedited blot and gel images

Supplemental tables 1-8

Supporting data values

## Figures and Tables

**Table 1:**
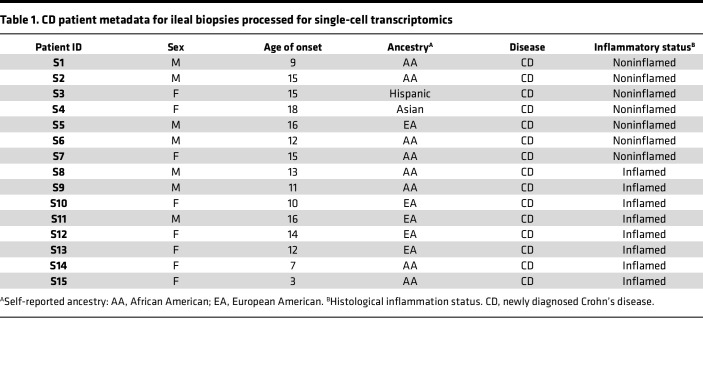
CD patient metadata for ileal biopsies processed for single-cell transcriptomics

**Table 2:**
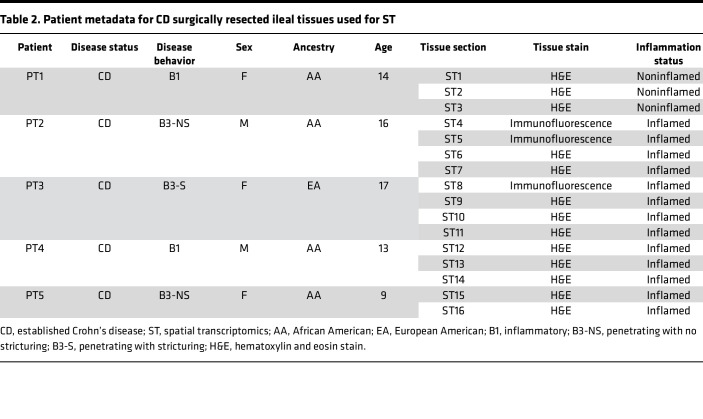
Patient metadata for CD surgically resected ileal tissues used for ST

## Version 1 03/10/2025

Electronic publication
