# Supplementary material for: Altered inflammatory mucosal signatures within their spatial and cellular context during active ileal Crohn’s disease
Source: JCI Insight. 2025 Mar 10;10(5):e171783. doi: 10.1172/jci.insight.171783 (PMC11949056; doi:10.1172/jci.insight.171783)

Supplementary Tables

Supplementary Table 1: Cell type marker genes used for manual cluster annotation

Supplementary Table 2: Differential gene expression results to define modules per cell type

Supplementary Table 3: Clustering results of differentially expressed genes into modules per cell type

Supplementary Table 4: Receptor-ligand interactions for non-inflamed and inflamed scRNA-seq data.

Supplementary Table 5: Pathways identified using non-negative matrix factorization of 10 factors per tissue in spatial transcriptomics data.

Supplementary Table 6: Overlapping pathways from single-cell and spatial transcriptomics methods.

Supplementary Table 7: Gene lists mapped onto spatial tissues from overlapping pathways in single-cell and spatial transcriptomics analyses.

Supplementary Table 8: Estimated cell type proportions per patient tissue section in antigen processing and CAM enriched spots.

Supplementary Figure Legends

Supplementary Figure 1. Data quality and pre-processing for single-cell transcriptomics. A. Schematic showing the pre-processing and quality control thresholds of the single cell data. **B.** Quality control metrics post elimination of ambient

RNA, low quality cells, high mitochondrial percentage cells and doublets. **C.** Expression of mucosal compartment marker genes in feature plots pre and post filtering. **D.** UMAP overlaying the three compartments post filtering. **E.** Cell type composition across samples. Percentage of cells on y-axis from each cell type.

Supplementary Figure 2. Enriched KEGG pathways in single cell transcriptomic differential expression modules A. Top 50 KEGG pathways across epithelial cell types. **B.** Top 50 KEGG pathways across immune cell types. Barplots include the disease pathways that had been filtered out in Figure 2B. **C**. Heatmap of all module genes from Figure 2E and their differential expression (inflamed vs non-inflamed) in all cell types.

Supplementary Figure 3. Reproducible cell-cell communication across the samples. A. Heatmap indicates the proportion of RL pairs (plus direction) conserved across individual samples (beginning with S) and groups (NI colored yellow and IF colored purple).

Supplementary Figure 4. Dysregulated ECM signaling in inflamed compared to noninflamed CD. Chord illustration of ECM receptor-ligand interaction in AGRN, HSPG, THBS, COLLAGEN and LAMININ pathways.

Supplementary Figure 5. Data quality and pre-processing of spatial transcriptomics dataset. A. Quality metrics of spatial transcriptomics processed samples before mitochondrial and ribosomal gene filtering. **B.** Quality metrics of spatial transcriptomics processed samples after filtering mitochondrial and ribosomal genes.

Supplementary Figure 6. Spatial non-negative matrix factorization demonstrates patient mucosal biology and compartmentalization. A. Mapping of three factors

corresponding to compartments (“compartment-based factors”) using spatial non-negative matrix factorization (left) and estimated cell type proportions using CARD deconvolution (right) in representative tissue section ST15 (from PT5). **B.** Hierarchical clustering of correlations between factors from non-inflamed PT1 (left) and inflamed PT2 (right) tissue sections.

Supplementary Figure 7. Enriched KEGG pathways across single-cell and spatial transcriptomic methodologies. Frequencies of all KEGG pathways enriched in both single cell transcriptomic modules and spatial transcriptomic factors, including disease and infection-related pathways that were filtered from Figure 5B.

Supplementary Figure 8. Cell type proportions deconvolved from ileal CD tissue sections. Hematoxylin and eosin staining of patient tissue sections and cell type deconvolution using CARD algorithm of two representative ST sections per patient, including ST2 and ST3 for Patient 1 (**A**), ST6 and ST7 for Patient 2 (**B**), ST10 and ST11 for Patient 3 (**C**), ST12 and ST13 for Patient 4 (**D**), and ST15 and ST16 for Patient 5 (**E**).

Supplementary Figure 9. Visualization of cellular localization and compositional changes in cell adhesion enriched tissue regions during Crohn’s disease.

A. For spatial tissues ST1 (Patient 1, non-inflamed) on top and ST14 (Patient 4, inflamed) on bottom. Plots from left to right represent hematoxylin and eosin staining (leftmost panel), overlay of cell adhesion molecule genes onto spatial tissues (second to leftmost), cell-type deconvolution of spatial tissues with expanded view of regions containing high enrichment of cell adhesion molecule genes as pies (second to right), and stacked barplot of 20 representative spots’ cell type proportions (right). **B.** Cell type co-localization corresponding to 20 representative highly enriched cell adhesion molecule spots in ST1

(Patient 1, non-inflamed) on left and ST14 (Patient 4, inflamed) on right. **C.** Immunofluorescence of activated macrophage markers ITGAX (red) and CD68 (green), epithelial marker E-cadherin (white), with DAPI (blue).

Supplementary Figure 1

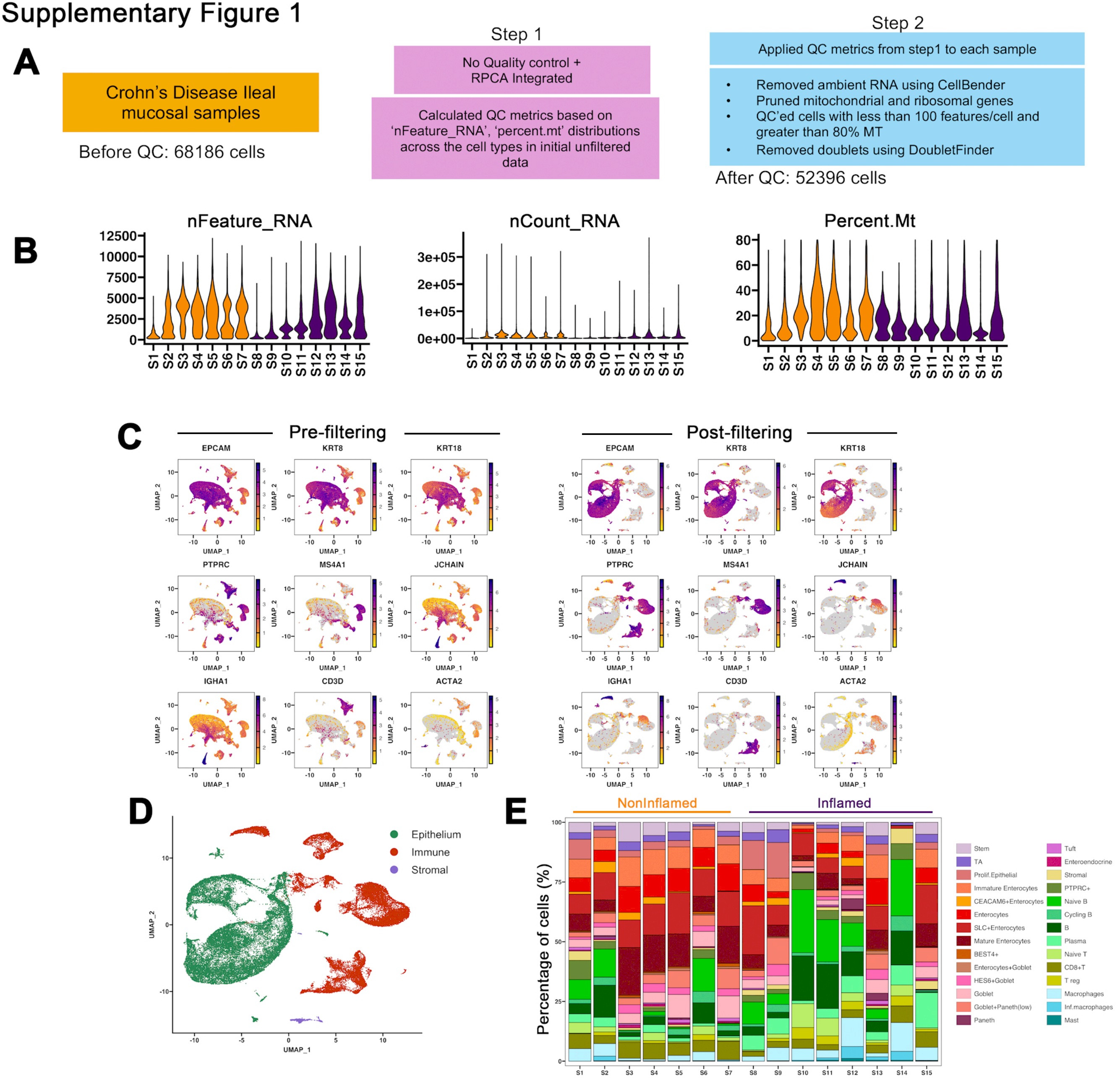

Supplementary Figure 2

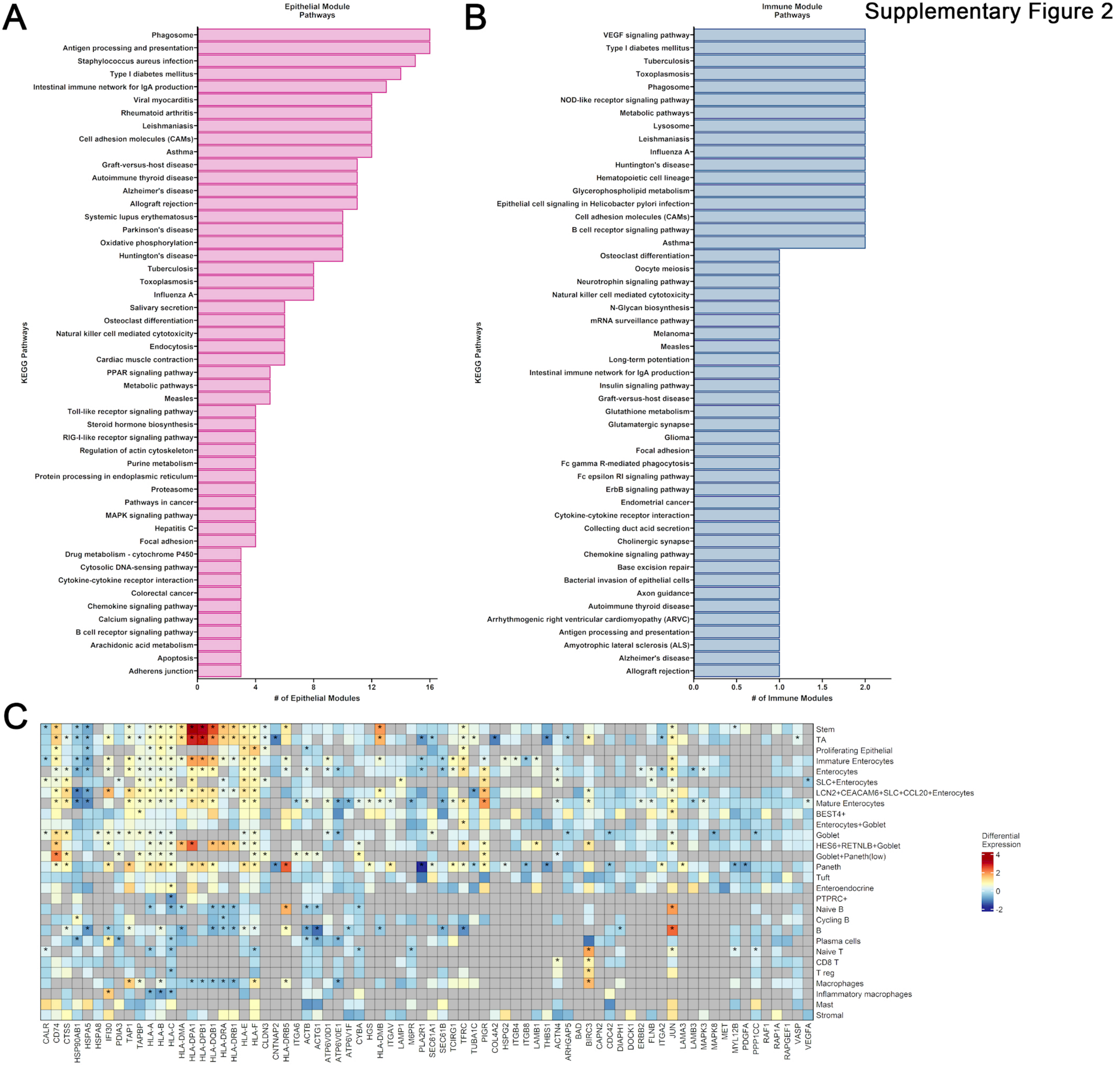

Supplementary Figure 3

A

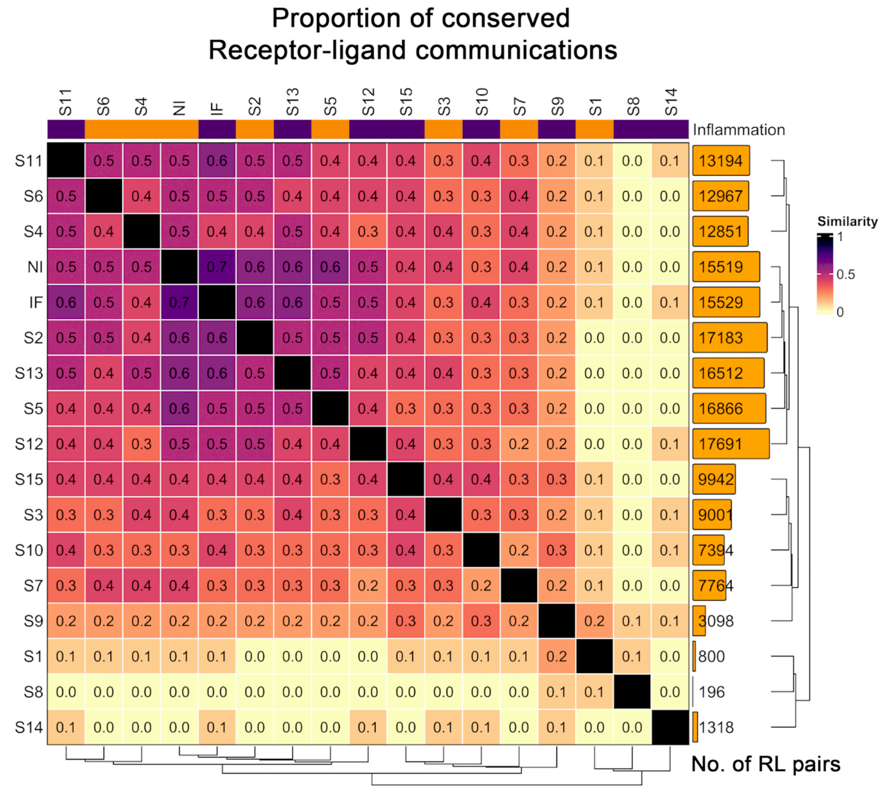

Supplementary Figure 4

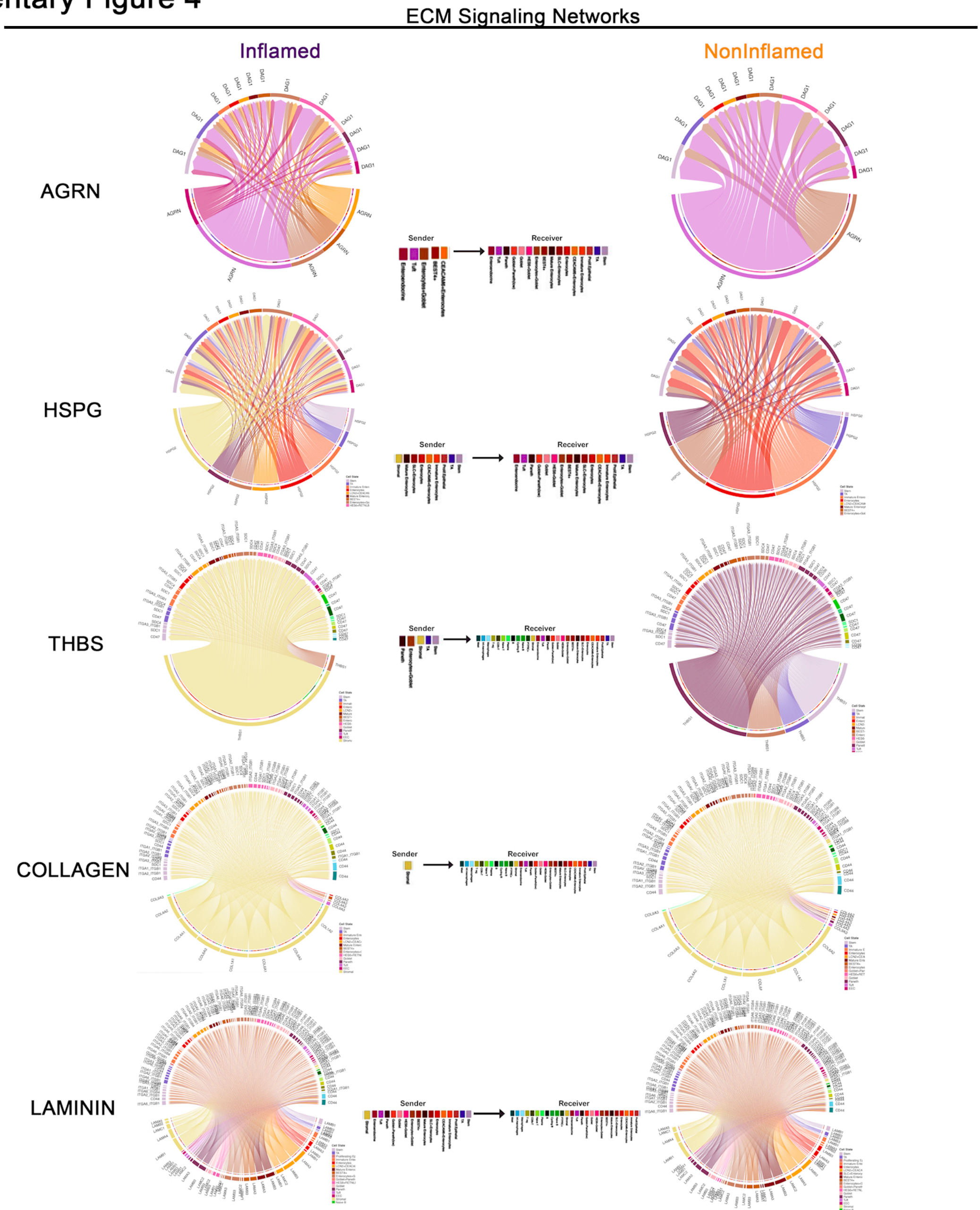

Supplementary Figure 5

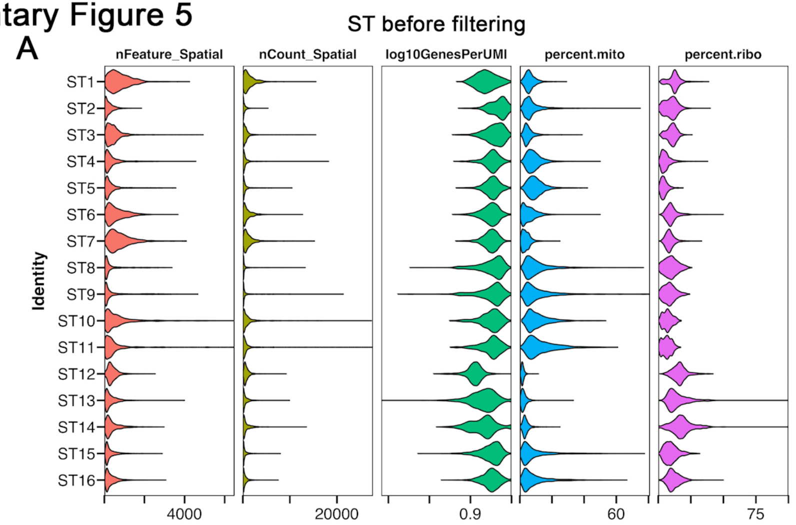

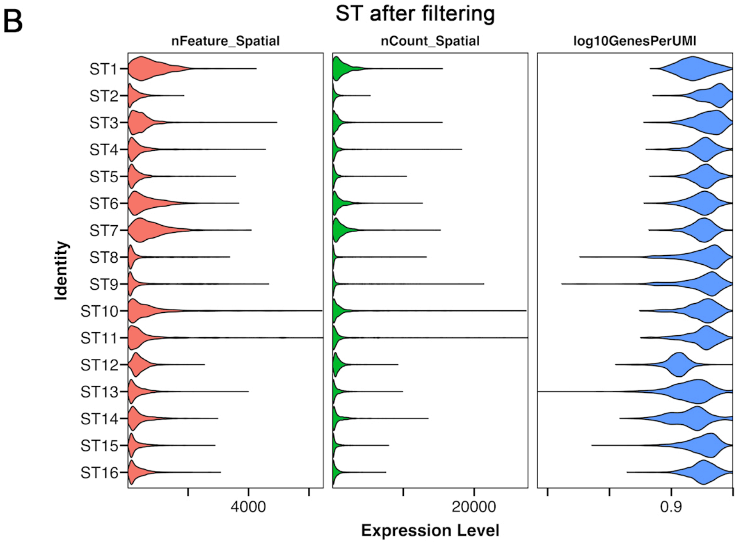

Supplementary Figure 6

A

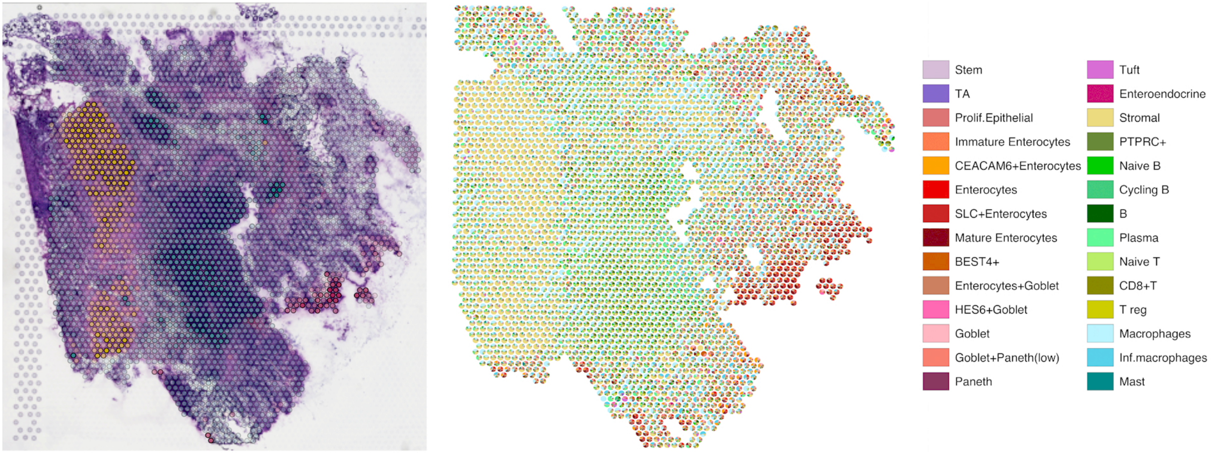

B

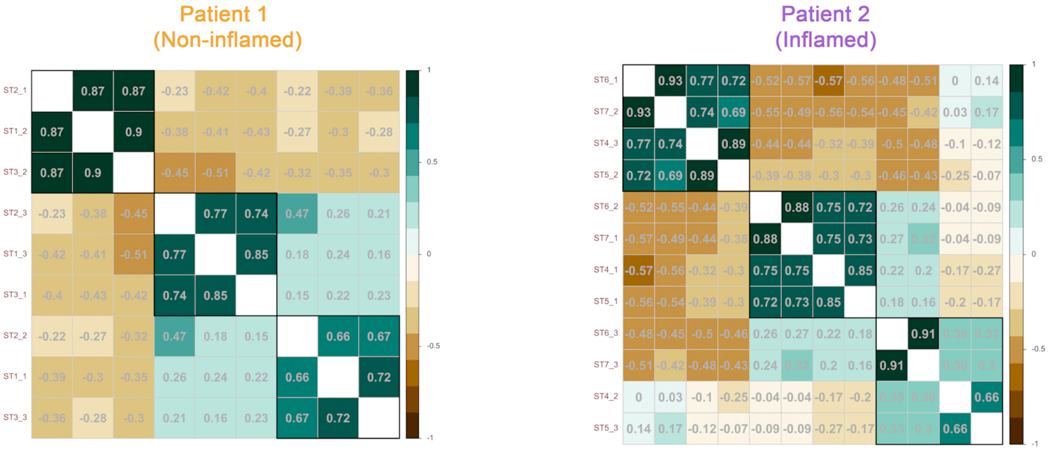

Supplementary Figure 7

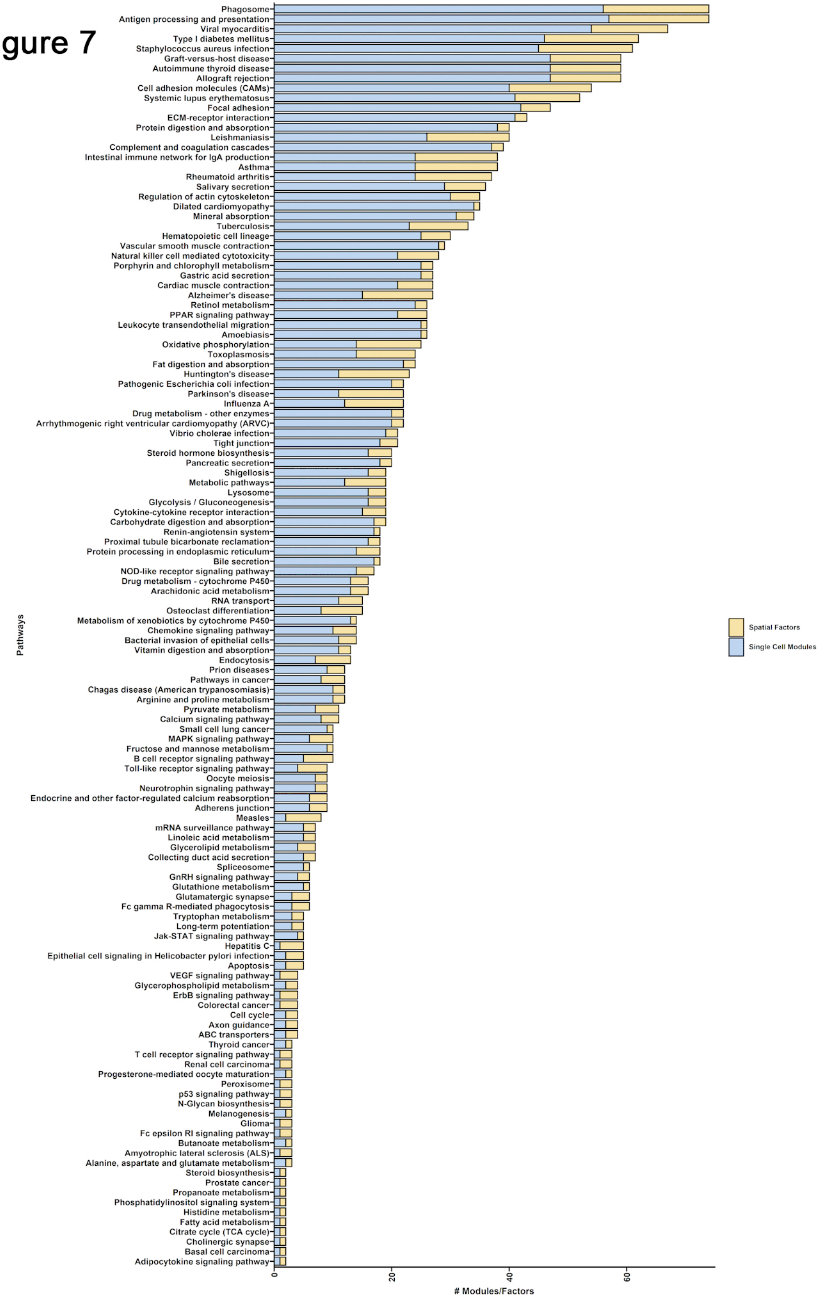

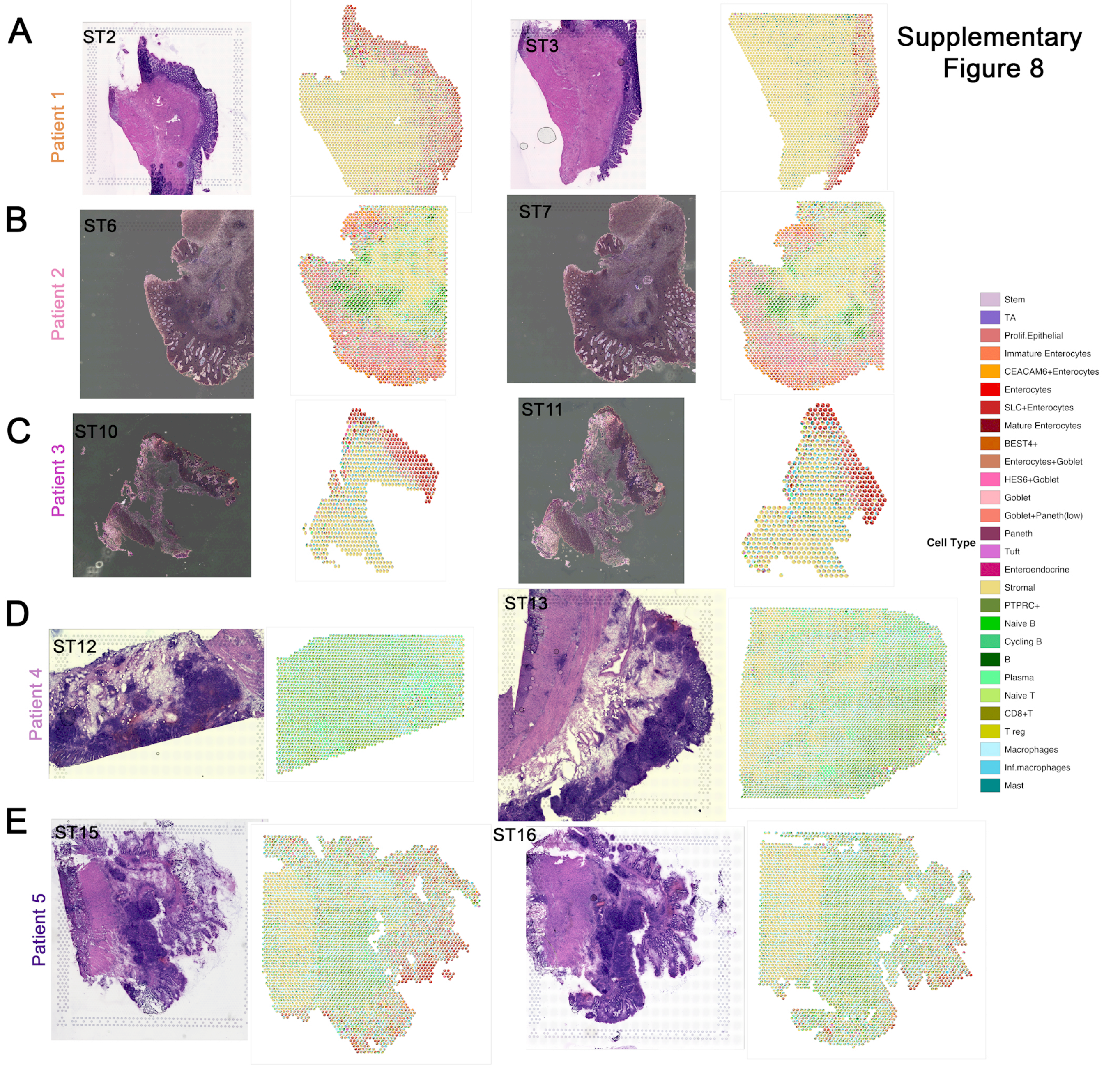
A
B
C
D
E
Patient 1
Patient 2
Patient 3
Patient 4
Patient 5
ST2
ST3
ST6
ST7
ST10
ST11
ST12
ST13
ST15
ST16
Supplementary
Figure 8
Cell Type
Stem
TA
Prolif.Epithelial
Immature Enterocytes
CEACAM6+Enterocytes
Enterocytes
SLC+Enterocytes
Mature Enterocytes
BEST4+
Enterocytes+Goblet
HES6+Goblet
Goblet
Goblet+Paneth(low)
Paneth
Tuft
Enteroendocrine
Stromal
PTPRC+
Naive B
Cycling B
B
Plasma
Naive T
CD8+T
T reg
Macrophages
Inf.macrophages
Mast

Supplementary Figure 9

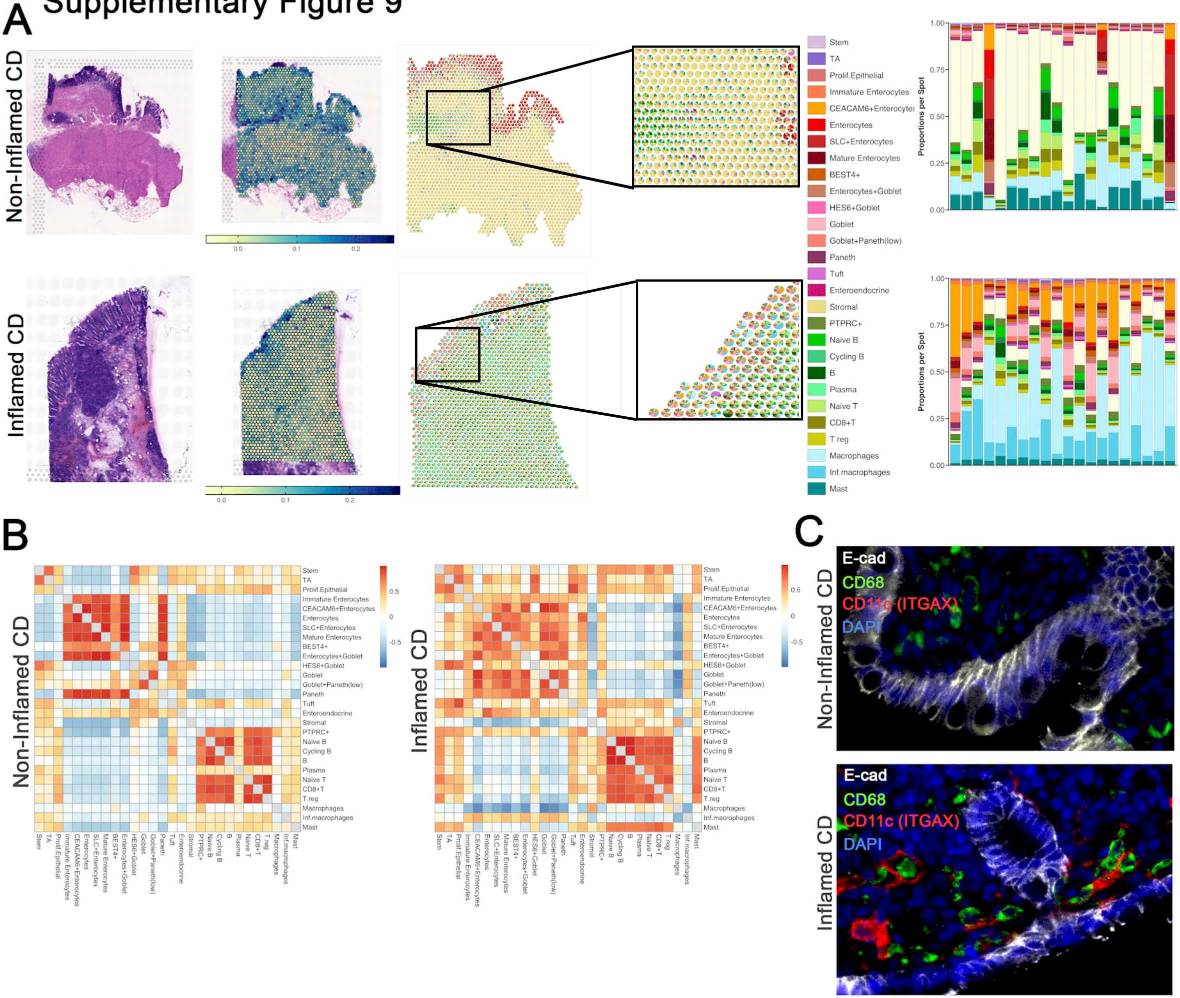

Supplement: Supplemental data [file jciinsight-10-171783-s271.pdf]
