# Supplementary figures and images for: Altered inflammatory mucosal signatures within their spatial and cellular context during active ileal Crohn’s disease
Source: JCI Insight. 2025 Mar 10;10(5):e171783. doi: 10.1172/jci.insight.171783 (PMC11949056; doi:10.1172/jci.insight.171783)

Unedited western blot of Figure 8E

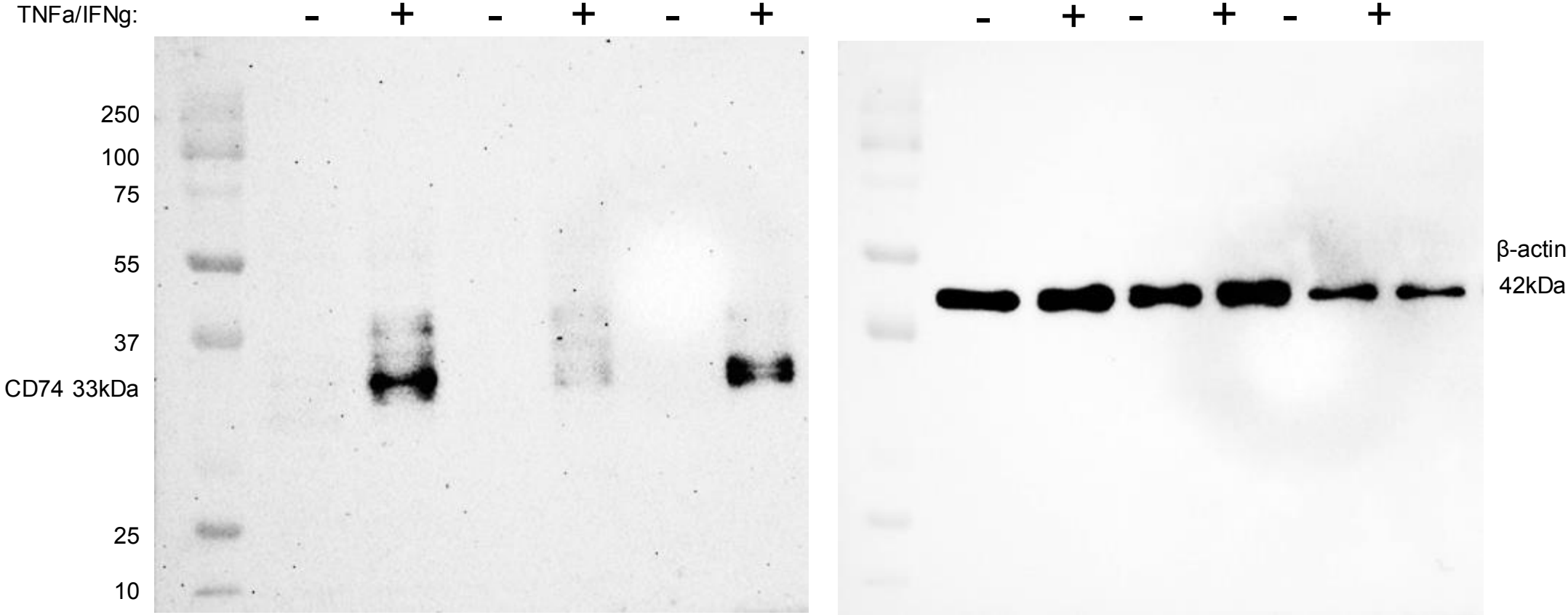

Supplement: Unedited blot and gel images [file jciinsight-10-171783-s272.pdf]
